# The Safety of Soy Leghemoglobin Protein Preparation Derived from *Pichia pastoris* Expressing a Soy Leghemoglobin Gene from *Glycine max*: *In Vitro* and *In Vivo* Studies

**DOI:** 10.1155/2023/7398724

**Published:** 2023-10-10

**Authors:** Teresa F. Reyes, Puja Agrawal, Teresa Chan, Richard Green, Ray A. Matulka

**Affiliations:** ^1^Impossible Foods Inc., 400 Saginaw Drive, Redwood City, CA 94063, USA; ^2^Burdock Group Consultants, 859 Outer Road, Orlando, FL 32814, USA

## Abstract

Soy leghemoglobin (LegH) protein derived from soy (*Glycine max*) produced in *Pichia pastoris* (reclassified *as Komagataella phaffii*) as LegH Prep is a novel food ingredient that provides meat-like flavor and aroma to plant-derived food products. The safety of LegH Prep has been previously assessed in a battery of *in vivo* and *in vitro* testing and found no adverse effects under the conditions tested. In this new work, we present the results of new *in vivo* and *in vitro* tests evaluating the safety of LegH Prep. LegH Prep was nonmutagenic in a bacterial reverse mutation assay and nonclastogenic in an *in vitro* micronucleus assay in human lymphocytes. Systemic toxicity was evaluated in the 90 day dietary study in male and female Sprague–Dawley® rats that included a 28 day recovery period. The study resulted in no animal deaths associated with the administration of LegH Prep at the highest dose (90,000 ppm). There were no significant adverse clinical or physical changes attributed to LegH Prep administration, and no observed adverse effects on either male or female rats over the course of the 28 day recovery phase study. The new 90 day dietary toxicity study established a no observed adverse effect level (NOAEL) of 4798.3 and 5761.5 mg/kg/day, the maximum level tested for male and female rats, respectively. Thus, the results of the studies demonstrate that under the conditions tested, LegH Prep is not toxic for consumption in meat analog products.

## 1. Introduction

Animal agriculture is one of the principal contributors to climate change, which through continuous emissions of greenhouse gases places enormous stress on Earth's land, water, and energy resources [1, 2]. One of the ways to combat the damaging environmental effects of animal agriculture is to harness the power of food biotechnology to develop novel alternative plant-based proteins thereby reducing dependence on animal agriculture's environmental footprint. The last decade has produced substantial advances in the field of alternative meat protein engineering. One of these has been the introduction of the soy leghemoglobin (LegH) protein. The characterization of LegH has been previously described [3, 4]. LegH protein is bioproduced by the yeast *Pichia pastoris* (currently reclassified as *Komagataella phaffi*) via industrial submerged fermentation. LegH Prep has a total protein fraction of at least 65% and is composed of LegH protein, *P. pastoris* yeast proteins, and food-grade stabilizers [4]. New toxicology data support the original safety assessment of this food ingredient derived from a novel source.

*Pichia pastoris* is a nontoxigenic and nonpathogenic microbial fermentation yeast used in the biomanufacturing of various FDA-notified GRAS substances [5–8], including LegH Prep derived from *P. pastoris* that obtained a “no questions” letter from FDA when a GRAS dossier was notified (GRN#737) describing use at levels of up to 0.8% soy LegH protein in a low number of different types of food products [9], similar to previous notified GRAS ingredients [8]. In 2019, FDA approval was achieved for soy LegH as a color additive [10]. LegH Prep derived from *P. pastoris* has been approved for use in food (meat analog products) in Australia-New Zealand [11], Singapore [12], and Canada [13], as well as meeting regulatory compliance in the United States. LegH Prep from *P. pastoris* has been used in the meat analog products produced by Impossible Foods since 2016 and has been sold and consumed internationally (over 500 million, ¼ pound (113 g) servings) without any significant reports of any safety issues concerning the consumption of soy LegH Prep.

The safety of LegH Prep has been previously assessed [4] for limited use conditions, using *in vivo*, *in vitro*, and *in silico* testing [14, 15]. The potential allergenicity and toxicity risk of LegH Prep was previously evaluated using bioinformatics, proteomics, and a pepsin digestion assay according to CODEX Alimentarius Commission [16] Guideline for the Conduct of Food Safety Assessment of Foods Produced Using Recombinant-DNA Microorganisms (CAC/Gl 46-2003 [15]). The previous published results demonstrated that the seven residual yeast proteins in LegH Prep (≥1% of the total protein content) displayed no significant sequence matches to any known food allergens except the highly conserved wheat glyceraldehyde-3-phosphate dehydrogenase (GAPDH) and similar alignment to homologous proteins from many common yeasts including *Saccharomyces* sp. [15]. Results published by Fraser et al. [4] demonstrated via a pepsin digestion assay that LegH and *P. pastoris* proteins were swiftly digested, thereby indicating that under the conditions of this assay, LegH Prep is not likely to pose an allergenicity risk. The authors concluded that there was no risk of cross-reactivity between LegH Prep and GAPDH. However, the published studies did not evaluate long-term administration of the LegH Prep in a preclinical study.

The safety of LegH Prep produced by *P. pastoris* (*K. phaffii*) for use in meat analog products at the maximum recommended application rates is supported by previously published toxicological data assessed in Sprague–Dawley® rats by a 28 day dietary study with an additional 28 day feeding study that evaluated female reproductive health and estrous cycle [4]. The mutagenic potential of LegH Prep was evaluated by a bacterial reverse mutation assay and potential genotoxicity in an *in vitro* chromosomal aberration assay. Overall, results from these *in vitro* and *in vivo* studies confirmed no issues of toxicological concern regarding LegH Prep under the conditions tested [4].

To expand the toxicological body of evidence attesting to the safety of LegH Prep and strengthen the assessment of safety under the intended conditions of long-term ingestion, Impossible Foods conducted new *in vivo* and *in vitro* studies to evaluate LegH Prep's potential for general and genetic toxicity. The *in vitro* models consist of a bacterial reverse mutation assay and an *in vitr*o micronucleus assay in human peripheral blood lymphocytes (HPBLs). Systemic toxicity was also evaluated via a 90 day dietary feeding study in Sprague–Dawley® rats that included a 28 day recovery period. Overall, the results of the studies in this new research underscore the lack of toxicological concern for LegH Prep under the conditions tested.

## 2. Materials and Methods

### 2.1. LegH Prep Production and Analysis

The soy plant (*Glycine max*) was the source of the LegH protein sequence which was inserted and expressed into a *P. pastoris* strain via submerged fed-batch fermentation and obtained using filtration-based methods and food and/or pharmaceutical-grade reagents. The *P. pastoris* production strains were derived from a nontoxigenic and nonpathogenic, safe strain lineage that has a history of safe use in the production of proteins for use in food and other applications [17, 18]. The LegH Prep used in the *in vivo* and *in vitro* studies (batches LH20-150-160-190FG-301 and PP-PGM2-20-061-302) met internal specifications. The strains were derived from the production strain MXY0291 [4, 6] and the well-known *Pichia* strain *NRRL Y-11430* [4, 19]. Both strains were engineered to overexpress the LegH gene as well as eight native enzymes in the *Pichia* heme biosynthesis pathway (aminolevulinic acid (ALA) synthase, ALA dehydratase, coproporphyrinogen oxidase, ferrochelatase, porphobilinogen deaminase, protoporphyrinogen oxidase, UPG III synthase, and uroporphyrinogen (UPG) III decarboxylase) [4]. Postfermentation, the cells were lysed to release the LegH protein. All insoluble materials were removed and the resulting concentrated liquid (LegH Prep) was formulated with food-grade stabilizers and frozen. To ensure that the test article was well incorporated in the animal diet, the LegH Prep was freeze-dried before use. All *in vivo* testing was performed at Product Safety Labs (PSLs), Dayton, New Jersey, USA. PSL has been accredited and certified by the Association for Assessment and Accreditation of Laboratory Animal Care (AAALAC). During the 90 day study and 28 day recovery period, the LegH protein concentrations in the neat test article and animal feed samples were analyzed by Impossible Foods using ultra-high-performance liquid chromatography (UHPLC) to assess LegH Prep concentration, homogeneity, and stability. UHPLC was performed using a Waters ACQUITY UHPLC system with an ACQUITY BEH SEC 4.6 × 150 mm column (Waters, Milford, MA). LegH protein concentration was quantified via integration of the 405 nm absorbance at the LegH retention time.

### 2.2. Bacterial Reverse Mutation Assay

The bacterial reverse mutation assay was performed by Eurofins (Munich, Germany) and was conducted in accordance with Good Laboratory Practice (GLP) regulations [20]. The study followed internal Eurofins Munich SOPs and the following guidelines: OECD Guidelines for Testing of Chemicals, Section 4, No. 471 [21], Commission Regulation (EC) No. 440/2008 B.13/14 [22], and the EPA Health Effects Test Guidelines, OCSPP 870.5100 [23].

Five bacterial strains *(Salmonella typhimurium* (ST) TA98 and TA1535 (Moltox, Inc., USA); TA100 and TA1537 (Xenometrix AG, Switzerland), and *Escherichia coli* (EC) WP2 *uvrA* (Moltox, Inc., USA)) were used in the plate incorporation and preincubation methods, in the presence and absence of a metabolic activation system (S9 mix, Trinova Biochem GmbH, Gießen, Germany). Sterile water was the negative control and vehicle (except where noted), while sodium azide (NaN_3_; Sigma), 4-nitro-o-phenylenediamine (4-NOPD; Sigma; dissolved in DMSO), methyl methanesulfonate (MMS; MilliporeSigma, USA), and 2-aminoanthracene (2-AA; Alfa Aesar, USA; dissolved in DMSO) were used as positive controls. LegH Prep cytotoxicity and the potential to induce mutations were assessed with tester strains TA98 and TA100 in a pre-experiment. Eight concentrations of LegH Prep were tested in triplicate under experimental conditions which were the same as those described below for the main experiment I (plate incorporation test). The LegH Prep concentrations utilized in the main experiments were chosen based on the results from the pre-experiment (S1, Supplementary Materials). As the pre-experiment results were in accordance with the criteria of validity (Supplementary Info Table S2), these results were reported as a part of the main experiment I.

The main experiment included an initial test that followed the plate incorporation method using the following materials which were combined and plated using minimal agar: 100 *μ*L of the prepared test solutions, negative (vehicle) control, or prepared positive control substance; 500 *μ*L S9 mix or substitution buffer; 100 *μ*L bacteria suspension (ST or EC); and 2000 *μ*L overlay agar [4]. After solidification, the plates were inverted and incubated at 37°C for 48 hours in the dark. The confirmatory test employed the plate incorporation method allowing for spacing between dose concentrations. Following incubation, revertant colonies were counted using a ProtoCOL counter (Meintrup DWS Labor Laborgeräte GmbH). The revertant colonies were counted manually if the precipitation of the test article precluded automatic counting. Low spontaneous mutation frequency tester strains TA1535 and TA1537 were counted by hand. Cell toxicity was identified by clearing or depletion of the background lawn or a reduction in the number of revertants down to a mutation factor of approximately ≤0.5 in relation to the solvent control. For the study to be considered valid, the bacteria (1) must have demonstrated typical responses to ampicillin, (2) the negative control plates (distilled water) with and without the S9 mix must be within the laboratory historical control ranges, (3) the corresponding background growth on both negative control and test plates must be visible, and (4) at least five different concentrations of each tester strain must be analyzable. For each experimental point, the mutation factor (MF) was calculated by dividing the mean value of the revertant counts by the mean values of the solvent control. LegH Prep would be considered mutagenic if a biologically significant positive response for at least one of the dose groups is observed in at least one tester strain with or without metabolic activation or if there is a clear and dose-related increase in the number of revertants.

A biologically significant positive response was scored if tester strains (TA98, TA100, and *E. coli* WP2 *uvrA*) resulted in twice as high the number of reversions and if tester strains TA1535 and TA1537 resulted in at least three times the number of reversions as compared to the control [24]. According to OECD guidelines, the biological relevance of the results serves as the criterion for the interpretation of results, and a statistical evaluation of the results is not regarded as necessary.

### 2.3. *In Vitro* Mammalian Micronucleus Assay in Human Peripheral Blood Lymphocytes (HPBLs)

The *in vitro* mammalian micronucleus assay was performed at Eurofins (Munich, Germany) in compliance with the German GLP regulations and under the appropriate OECD [25] and European Commission [26] guidelines. The study employed human peripheral blood lymphocytes (HPBLs) in both the absence and the presence of chemically induced rat liver S9 metabolic activation system (either prepared at Eurofins Munich or obtained from Trinova Biochem, Giessen, Germany). Blood was collected from a single donor with no known recent exposure to genotoxic chemicals, or radiation to reduce interindividual variability and samples was stored in heparinized tubes at 4°C for a maximum of 4 hours. Whole blood samples treated with heparin were precultured in the presence of mitogen (phytohemagglutinin, PHA). HPBLs were cultured in complete medium (RPMI 1640 containing 15% heat-inactivated fetal bovine serum (FBS), 2.4 *µ*g/mL of phytohemagglutinin, and 100 units of penicillin/streptomycin solution). A pre-experiment was conducted under identical conditions described for the main experiment I (4 hour incubation) to determine the cytotoxicity of the LegH Prep using the cytokinesis-block proliferation index (CBPI). The following concentrations were tested with or without S9 mix: 10, 20, 39, 78, 156, 312.5, 625, 1250, 2500, and 5000 *µ*g/mL. The LegH Prep was suspended and diluted in cell culture medium (RPMI) within 1 hour prior to treatment. After ultrasonication for 30 minutes at room temperature, a stable suspension was obtained. The pH value was within a physiological range in the test item. All positive control substances used were from Sigma unless specified. Methyl methanesulfonate (MMS, 50 and 65 *µ*g/mL—without metabolic activation) and cyclophosphamide (CPA, 15 *µ*g/mL15 *µ*g/mL—with metabolic activation) were used as clastogenic controls and colchicine (0.04 and 0.4 *µ*g/mL) (without metabolic activation) was used as an aneugenic control.

#### 2.3.1. Experiment I (Metabolic Activation)

Whole blood samples were precultured (44 to 48 hours) in the presence of PHA prior to LegH Prep dosage. LegH Prep was added to the lymphocytes then incubated for 4 hours in the presence or absence of metabolic (S9) activation. The treatment medium (complete culture medium without FBS) was removed at the end of the incubation period; the cells were then washed and the cultures were incubated in complete culture medium + 6 *µ*g/mL cytochalasin B for 40–42 hours at 37°C [27].

#### 2.3.2. Experiment II (No Metabolic Activation)

Whole blood cultures were precultured in the presence of PHA for 44 to 48 hours prior to exposure to LegH Prep and were added in a complete culture medium. An hour later, 6 *µ*g/mL cytochalasin B was added and the cells were incubated for a further 43 hours at 37°C. The culture medium was removed at the end of the treatment period, and the cells were prepared for microscopic analysis. Duplicate cultures were analyzed at each dose level except for the pre-experiment.  Table 1 outlines the study design.

#### 2.3.3. Culture Preparation

At the end of incubation, the complete culture medium was removed, and the cells treated with a cold hypotonic solution (0.075 M potassium chloride) at room temperature then centrifuged. The pellet was resuspended with a fixation solution and centrifuged. The collected cells were fixed with a methanol (3 parts) + glacial acetic acid (1 part) solution, resuspended, and loaded onto clean glass slides and dried and stained with acridine orange solution.

#### 2.3.4. Analysis of Micronuclei

For each dose group, at least 2000 binucleated cells (if possible) per concentration (1000 binucleated cells per slide) were analyzed for micronuclei [28]. Mononucleated and multinucleated cells and cells with more than six micronuclei were not considered [29].

#### 2.3.5. Cytokinesis-Block Proliferation Index (CBPI)

To properly evaluate cytotoxicity, a cytokinesis-block proliferation index (CBPI) was determined from 500 cells according to the following formula:

(1) $$\begin{array}{c} \text{CBPI}=\frac{\left(c_{1}\times 1\right)+\left(c_{2}\times 2\right)+\left(c_{x}\times 3\right)}{n}, \end{array}$$

where *c*_1_ is mononucleate cells, *c*_2_ is binucleate cells, *c*_*x*_ is multinucleate cells, and *n* is the total number of cells.

The CBPI was used to calculate the % cytostasis, which indicates the inhibition of cell growth of treated cultures in comparison to control cultures:

(2) $$\begin{array}{c} \%\text{ Cytostasis}=100\;-\;100\times \left[\frac{\left({\text{CBPI}}_{T}-1\right)}{\left({\text{CBPI}}_{C}-1\right)}\right], \end{array}$$

where CBPI_*T*_ is the cytokinesis-block proliferation index of treated cultures and CBPI_*C*_ is the cytokinesis-block proliferation index of control cultures.

#### 2.3.6. Statistical Analysis

Significance was decided at a probability value of *p* < 0.05. The nonparametric *χ*^2^ test was performed to analyze the results in both experiments.

### 2.4. 14 Day Dietary Toxicity/Palatability Study in Rats

The 14 day toxicity/palatability study followed OECD Guidelines 407 [30] and was compliant with US FDA guidelines [31]. The protocol for this *in vivo* study was preapproved (P700) by the IACUC of the laboratory (Dayton, NJ), and the laboratory has been accredited by the AAALAC organization. Sprague–Dawley CD® IGS rats from Charles River Laboratories (Kingston, New York) were obtained and placed under quarantine during acclimation for five days. While separate processes, quarantine and acclimation commence concurrently. The animals are analyzed for signs or symptoms of unknown pathogens, and designated (sentinel) animals undergo blood collection for viral screening to confirm the absence of common viruses to Sprague–Dawley rats. As a prerequisite for experimental conduct, the animals are acclimated to the test facility for an appropriate amount of time, outlined in regulatory guidelines. The rats were kept in a temperature- and humidity-controlled room at 19°C to 21°C and 58% to 85%, respectively, under a 12 hour light-dark cycle and fed a standard Certified Envigo Teklad Global Rodent Diet (Envigo Laboratories, Inc., Indianapolis, IN.). The diet and filtered tap water were supplied *ad libitum*. All contaminants measured in the diet and filtered tap water were within acceptable regulatory standards. The rats were individually housed and received object enrichment (Nylabone® or IChew) throughout the study duration. Forty animals were selected for the test (7 weeks of age at dosing; 20 males (192–242 g) and 20 nonpregnant, nulliparous females (166–204 g) were randomly assigned into 4 groups (*N* = 5/sex/group). The freeze-dried LegH Prep was administered in the diet at concentrations (0, 50000, 100000, and 150000 ppm) that targeted 0 (Group 1, control), 4167 (Group 2), 8333 (Group 3), and 12,500 (Group 4) mg/kg/day LegH Prep (Supplementary Table S3). Stability of the neat LegH Prep was maintained while under storage conditions at PSL over the course of the study period. Homogeneity and dietary stability analyses showed that LegH Prep was homogeneously distributed and was stable in the dietary matrix (data not shown). Individual animal food consumption was determined simultaneously alongside body weight measurements. The animals were checked at least twice a day for any sign of toxicity, survivability, and comportment. Animal body weight was recorded twice, first during acclimation at receipt of the animals and on Day 0 and on test Days 3, 7, 10, and 14. Bodyweight gain was calculated for weekly intervals and for the overall study. Blood was collected from fasted (overnight) animals via the sublingual vein or vena cava/abdominal aorta, under isoflurane anesthesia. The collected blood (∼1 mL) was centrifuged (refrigerated) and the resulting serum was stored at −80°C in a preservative-free tube until clinical chemistry analysis. Whole blood samples (stored under refrigeration) were analyzed on an ADVIA 120 hematology system, and the following hematological parameters were evaluated: hematocrit (HCT), hemoglobin concentration (HGB), mean corpuscular hemoglobin (MCH), mean corpuscular volume (MCV), platelet count (PLT), red blood cell (RBC) count, red cell distribution width (RDW), reticulocyte (RET) count, white blood cell (WBC), and differential leukocyte count. Mean corpuscular hemoglobin concentration (MCHC) was calculated. In addition, serum clinical chemistry parameters were evaluated on a COBAS C311 automated analyzer, which included albumin (ALB), alkaline phosphatase (ALKP), bilirubin (BILI total), blood creatinine (CREA), calcium (CALC), chloride (CL), cholesterol (CHOL total), fasting glucose (GLU), globulin (GLOB), inorganic phosphorous, lipoprotein (high and low density), potassium (K), serum alanine aminotransferase (ALT), serum aspartate aminotransferase (AST), serum protein (total), sodium, sorbitol dehydrogenase (SDH), triglycerides (TRIG), blood urea nitrogen (BUN), and creatine phosphokinase (CPK). Separate blood smears were prepared from each animal undergoing hematological evaluation and, if necessary, were stained with Wright–Giemsa stain and examined to substantiate or clarify the results of hematological findings. Terminal sacrifice was performed by exsanguination under isoflurane anesthesia. All animals in the study underwent a gross necropsy, which entailed a detailed assessment of the animal's physical appearance, body orifices, the musculoskeletal system, and organs associated with the cranial, thoracic, abdominal, and pelvic cavities.

### 2.5. 90 Day Dietary Palatability Study in Rats with a 28 Day Recovery Study

The 90 day dietary palatability study in CRL Sprague–Dawley CD® rats with a 28 day recovery period was conducted at PSL according to GLP and OECD guidelines [32] and the U.S. FDA guidelines [31]. The protocol for this *in vivo* study was preapproved (P703) by the IACUC of the laboratory (Dayton, NJ), and the laboratory has been accredited by the AAALAC organization. The animals were quarantined/acclimated at PSL (as described in Section 2.4) for five days before the study starts. All animals were individually housed and provided a form of object (Nylabone® or IChew) enrichment. Animal body weight and clinical observation data were recorded at least twice before the study starts. Typical of protocols for studies completed for regulatory evaluations, including the European Chemicals Agency (ECHA) [33], four groups of adult CRL Sprague–Dawley CD® IGS rats (10/sex/group) were maintained on diets prepared to target daily intakes of 1875, 3750, and 5625 mg/kg/day LegH Prep for Groups 2–4, respectively (Supplementary Table S4), with five additional animals from Groups 1 and 4 remained on the study for an additional 28 day recovery period (Supplementary Figure S1). Rats in the control group were provided diet that was consistent with diets with the other groups but did not contain LegH Prep. Diet information is provided as a supplement to this manuscript (Supplementary Table S5). The neat LegH Prep was monitored for stability throughout the study and was found to be stable. Homogeneity and dietary stability analyses showed that LegH Prep was homogeneously distributed and stable in the dietary matrix during a 4 day preparation interval (data not shown). The dietary concentrations to provide 3750 and 5625 mg/kg bw/day were considered to have met the target concentrations. All animals received an ophthalmological evaluation by focal illumination and slit lamp biomicroscopy, prior to study initiation, and for all animals on Day 87. All animals were observed once a day for any sign of toxicity, survivability, and behavior and weekly for detailed clinical observations. Body weights were recorded twice during acclimation, including prior to test initiation on Day 0, and weekly thereafter until Day 91 (main test) and on Day 119 (recovery). Food consumption measurements were taken to coincide with body weight measurements. Food efficiency and dietary intake were calculated. Clinical pathology and a thyroid hormone assessment were performed on both the main test and the recovery phase animals. Urine samples (utilizing metabolism cages) and blood samples (via sublingual bleeding under isoflurane anesthesia) were collected on Days 92/120 for males and Days 93/120 for females for the main test and recovery animals, respectively. Blood was collected (∼500 *μ*L) in a precalibrated tube containing K_2_EDTA for hematological tests. The whole blood samples were centrifuged (refrigerated) and approximately 1 mL of serum was collected into a preservative-free tube for serum chemistry tests utilizing the COBAS C311 automated analyzer. Hematological analysis completed on an ADVIA 120 Hematology System included RBC, HCT, MCV, MCH, Absolute ARET, WBC, differential leukocyte count, MCHC, hemoglobin (HGB), MCV, RDW, and PLT. Coagulation analysis on a Siemens Sysmex CA620 system included prothrombin time (PT) and activated partial thromboplastin time (APTT). Clinical chemistry determined on a COBAS C311 analyzer included AST, SDH, BILI total, CREA, TRIG, total serum protein, globulin (GLOB), inorganic phosphorus, K, ALT, ALKP, BUN, total CHOL, GLUC, ALB, CALC, NA, and CL. Urinalysis included quality, color (COL), clarity, urine volume (UVOL), microscopic urine sediment examination, pH, GLUC, specific gravity, protein (UMTP), ketone, bilirubin, blood, and urobilinogen. Study animals were euthanized under isoflurane anesthesia, and blood was collected for evaluation of coagulation parameters. Vaginal smears were collected from all female rats on the day of terminal sacrifice (day 93 for the main test animals and day 120 for the recovery phase animals) to determine the stage of estrus. Gross necropsies were performed on all animals, and histological evaluation of selected organs and tissues was performed on Groups 1 and 4. All clinical pathology sample analyses were performed at PSL (Dayton, NJ). Study animals underwent a gross necropsy, which entailed a detailed assessment of the animal's physical appearance, body orifices, the musculoskeletal system, and all organs associated with the cranial, thoracic, abdominal, and pelvic cavities. Tissues and organs were collected and preserved in 10% neutral-buffered formalin except for the eyes, testes, and epididymis, which were preserved in Davidson's fixative before utilizing a gradient transfer process, with final storage in absolute ethanol prior to shipment of tissues to the pathology lab for histology processing. A subset of tissues/organs was weighed wet immediately after dissection to avoid desiccation including adrenal glands, kidneys, spleen, brain, liver, thymus, testes, epididymis, ovaries with oviducts, uterus, and heart. All preserved animal tissues were sent to StageBio (Mount Jackson, VA) for further processing and analysis by a board-certified veterinary pathologist. Tissues from all the main study animals in the control and high dose group, the female reproductive organs (from all dose levels), as well as all gross lesions from all animals were processed, embedded in paraffin, sectioned, and stained with hematoxylin and eosin (H&E).

#### 2.5.1. Statistical Analysis—14 and 90 Day Dietary Feeding Study in Rats

Statistical analysis on all the data collected during the in-life phase of both the 14 and 90 day studies was performed by PSL. The probability value of *p* < 0.05 was set for significance. The mean and standard deviation were calculated for all quantitative data. Male and female rats were evaluated separately. Statistical analysis was performed on all quantitative data for in-life and organ weight parameters using Provantis™ version 10, tables and statistics, Instem LSS, Staffordshire, UK. For the 14 day study, the following programs were used for analysis INSTAT or Prism Biostatistics, GraphPad Software, San Diego, CA; Statview, version 5, SAS Institute Inc., Cary, NC; and SigmaStat, version 2, SYSTAT Software, San Jose, CA. For the 14 day study, a two-way analysis of variance (ANOVA) was used to compare all in-life endpoints in both treatment and control groups that were classified as having multiple measurements of continuous data over time (e.g., body weight parameters, food consumption, and food efficacy), thereby testing the effects of both time and treatment, with methods accounting for repeated measures in one independent variable (time) [34]. Groups where variance is found to be significantly different were compared using a nonparametric method such as the Kruskal–Wallis nonparametric analysis of variance. If a nonparametric ANOVA was significant, a comparison of treated groups to control was performed (e.g., Dunn's test). If warranted by sufficient group sizes, the incidence of clinical observations may be evaluated through sequential application of a trend test [35].

For the 90 day study, the following parameters were calculated and analyzed by the Bartlett test for homogeneity of variances and normality [36]: body weights, food consumption, UVOL, hematology, blood chemistry, absolute and relative organ weights, averages, and standard deviations. One-way analysis of variance (ANOVA) was used to compare treated and control groups and the Bartlett test indicated homogenous variances. When ANOVA was significant, a comparison of the treated groups to control by the Dunn test for multiple comparisons was performed [37, 38]. Where variances were considered significantly different by the Bartlett test, groups were compared using a nonparametric method (Kruskal–Wallis nonparametric ANOVA) [39]. When nonparametric ANOVA was significant, a comparison of treated groups to control was performed using the Dunnett test [40]. Clinical pathology was preliminarily tested via the Levene test [41] for homogeneity and via the Shapiro–Wilk test [42] for normalcy followed by ANOVA and the Dunnett test [37, 38].

## 3. Results

### 3.1. Bacterial Reverse Mutation Assay

The pre-experiment analysis found no limiting toxicity nor limiting precipitation of the test item was observed in either tester strain used at the maximum recommended concentration of 5000 *µ*g/plate (with and without metabolic activation; Supplementary Table S1). Therefore, concentrations of 31.6 to 5000 *µ*g/plate were selected for the main experiments. Data results for Experiments I and II are shown in Tables 2–5.

In Experiment I, LegH Prep precipitation was observed in tester strains TA98 and TA100 at ≥1000 *µ*g/plate (with and without metabolic activation), and in tester strains TA1535, TA1537, and *E. coli* WP2 uvrA at ≥316 *µ*g/plate (with and without metabolic activation) (Table 2). In Experiment II, precipitation was observed in all tester strains at ≥316 *µ*g/plate (with and without metabolic activation) (Table 4). The observed precipitation did not interfere with the scoring; thus, it did not impact the results. In both experiments the mutation factors were within typical ranges (Tables 3 and 5). Four plates in Experiment I (TA100, 2500 *µ*g/plate; TA1535, 31.6 *µ*g/plate; TA1537, 5000 *µ*g/plate; *E. coli* WP2 *uvrA*, 5000 *µ*g/plate; without metabolic activation) exhibited microbial contamination but did not affect the quality, integrity, or evaluation of the results as the microbial contamination could be clearly distinguished from the tester strain revertants. In Experiment I, cytotoxic effects of LegH Prep were observed in tester strain TA1535 at 5000 *µ*g/plate (without metabolic activation) (Table 2). In Experiment II, cytotoxic effects of the test item were noted in tester strain TA1537 at ≥2500 *µ*g/plate (without metabolic activation) (Table 4). No further cytotoxic effects of the test item were noted in Experiments I or II. No biologically relevant increases in revertant colony numbers of any of the five tester strains were observed following treatment with LegH Prep at any concentration level neither in the presence nor in the absence of metabolic activation in Experiments I and II.

### 3.2. *In Vitro* Mammalian Micronucleus Assay in Human Lymphocytes

The potential of LegH Prep to induce micronuclei in human peripheral blood lymphocytes (HPBLs) in the absence and presence of metabolic activation with S9 was evaluated. The concentrations used in the main experiments (I and II) were based on the pre-experiment (Supplementary Table S6), with precipitation of LegH Prep observed in the pre-experiment at ≥312.5 *µ*g/mL with and without metabolic activation. LegH Prep was analyzed at 650 and 250 *µ*g/mL with and without metabolic activation, respectively, in Experiment I. In Experiment II, 750 *µ*g/mL was selected as the highest concentration (with and without metabolic (S9) activation) for microscopic analysis of micronuclei. The concentrations evaluated for micronuclei frequencies are provided in Table 6.

LegH Prep precipitation at the end of treatment was observed at ≥650 *µ*g/mL without metabolic activation and at ≥250 *µ*g/mL with metabolic activation in Experiment I and at ≥750 *µ*g/mL in Experiment II (Tables 7 and 8). No increase in the cytostasis above 30% was observed in both experiments and no biologically relevant increase of the micronucleus frequency was noted after treatment with LegH Prep. No statistically significant increase (*p* < 0.05) of cells with micronuclei by LegH Prep was noted in either Experiment I or Experiment II with and without metabolic activation. No statistically significant increase in the frequency of micronucleated cells under the experimental conditions of the study was observed in both Experiments I and II. The clastogenic positive controls methyl methanesulfonate (MMS, 50 and 65 *µ*g/mL) and cyclophosphamide (CPA, 15 *µ*g/mL) were used, and colchicine (0.04 and 0.4 *µ*g/mL) was the aneugenic control; all positive controls induced statistically significant increases in the micronucleus frequency.

### 3.3. 14 Day Dietary Toxicity/Palatability Study in Rats

The LegH Prep was added to the feed to administer target doses of 4167, 8333, and 12,500 mg/kg/day (Supplementary Table S4). The feed formulation was held constant throughout the study. LegH Prep remained stable and homogenous throughout the study (data not shown). Mean dietary intakes were calculated to be 4646.9, 8843.5, and 13035.9 mg/kg/day for males and 4175.9, 8686.0, and 12401.5 mg/kg/day for females, respectively.

No mortalities and no changes in mean body weight (Table 9), LegH Prep intake (Table 10), mean daily body weight gain, food consumption, and food efficiency (Supplementary Tables S7–S9) that were ascribed to the administration of LegH Prep occurred during the 14 day study. In-life clinical signs were comparable between the control and LegH Prep dose groups.

#### 3.3.1. Pathology

Dietary exposure to LegH Prep for 14 days in both male and female rats did not induce any biologically adverse changes in hematology and clinical chemistry parameters. Significant increases in mean phosphorus levels in Group 4 males and potassium levels in Group 3 and Group 4 males from control Group 1 were observed. All the changes in hematology parameters were considered unrelated to LegH Prep administration, including those that attained statistical significance because they occurred sporadically and were considered unrelated due to biological variance among rats as the magnitude of variation was minimal. Clinical chemistry parameters for male and female rats in Groups 2–4 were generally comparable to control Group 1 throughout the study except for statistically significant decreases (*p* < 0.05–0.01) in mean phosphorus levels for Group 4 males and in potassium levels for Groups 3 and 4; see Tables 11 and 12 for pathology results showing summary tables describing the mean, hematology, and clinical chemistry results. The significant potassium and phosphorus results were within historical control ranges and the control levels for these parameters were on the very low end of the historical control range for the laboratory and rat strain, which resulted in a statistically significant response but was not a biologically significant effect. The nonsignificant increase in the female Group 4 AST value was not considered toxicologically significant, as there was a high degree of variability in the results indicating potential issues with the samples and not a consistent, toxicology-related effect.

### 3.4. 90 Day Dietary Study in Rats with a 28 Day Recovery Period

The neat LegH Prep was monitored for stability and deemed stable over the course of the study to within an acceptable margin of variability. Homogeneity and dietary stability analyses showed that LegH Prep was evenly distributed and was stable in the dietary matrix during the 4 day preparation interval. Homogeneity analysis of Day 0 dietary preparations reported a relative standard deviation of 2.82, 1.12, and 0.19% for dietary concentrations of 30,000, 60,000, and 90,000 ppm dose group formulations. Stability testing found that the test substance was at 90.4, 95.7, and 99.5% at Day 4 of nominal concentrations of 30,000, 60,000, and 90,000 ppm of the LegH Prep for Groups 2–4, respectively. The dietary concentrations of 60,000 and 90,000 ppm, the intermediate and highest levels tested, were considered to have met target concentrations. Week 13 concentration verification for 30,000 ppm was below target.

**Figure 1:**
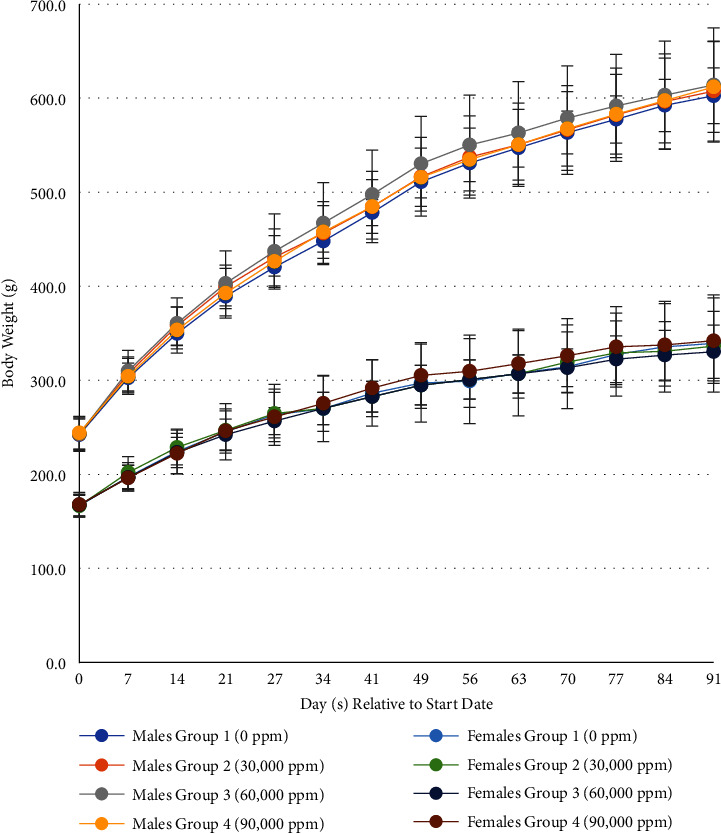
Summary of mean weekly body weights (g) 90 day dietary study^a,^^*∗*^, ^a^one-way repeat ANOVA and Dunnett test, ^*∗*^*N* = 15/sex/group for groups 1 and 4 (includes recovery animals), and *n* = 10/sex/group for groups 2 and 3.

No mortalities occurred over the course of the study. There were no clinical observations attributed to the dosing of LegH Prep. All clinical observations noted were considered incidental and of no toxicological relevance, as there were no trends in observations that increased with the dietary level. Also, there were no changes in body weight (Figure 1, Tables 13 and 14), body weight gain (data not shown), food consumption, or food efficiency of male and female rats over the course of this main study phase or recovery phase attributed to the dietary intake of LegH Prep (Supplementary Tables S10–S13). The daily intake of LegH Prep was calculated by body weight and food consumption measurements collected over the course of the study. Mean weekly body weights and mean daily body weight gains for male and female rats in Groups 2–4 (30,000–90,000) were comparable to control Group 1 (0 ppm) throughout the 90 day study and recovery period. The mean overall (Days 0–91) daily intake for the main test rats fed 30,000, 60,000, and 90,0000 ppm of the LegH Prep was calculated to be 1637.3, 3202.3, and 4820.4 mg/kg/day of LegH Prep for males and 2024.8, 4127.9, and 5930.8 mg/kg/day LegH Prep for females, respectively (Table 15). These values were generally in good agreement with targeted LegH Prep exposure concentrations in mg/kg/day for males and females.

#### 3.4.1. Pathology

Dietary administration of LegH Prep for a period of at least 90 days in male and female rats, at target dietary levels of 30,000, 60,000, and 90,000 ppm, did not result in any adverse effects on terminal body weights, organ weights, or clinical parameters such as hematology, coagulation, clinical chemistry, thyroid hormones, and urinalysis parameters during either the main study or recovery phases (Tables 16–23). All statistically significant changes were within historical control ranges, without histopathological correlate, and were not considered adverse.

A slight increase in LDL cholesterol in the intermediate dose (60,000 ppm male group) was considered toxicologically insignificant as there was no dose progression (Table 19). Dietary exposure to LegH Prep at levels of up to 90,000 ppm for at least 90 days resulted in no test article-related macroscopic observations, organ weight changes, or microscopic findings. Significant changes in absolute (Tables 16 and 17) and relative thymus weight, as well as epididymis-to-body weight, and significant increases in kidney-to-brain weights were observed in a nondose-dependent manner and were therefore not considered related to LegH Prep consumption (Tables S14 and S15). No significant alterations were found in the estrous cycle distribution between the control and high dose groups during the main phase or recovery phase of the 90 day study (Supplementary Table S16).

## 4. Discussion/Conclusion

Impossible Foods developed an innovative approach to bring change to the alternative protein market via the discovery of soy leghemoglobin's unique organoleptic properties which mimic the taste and aroma of animal meat. By replacing animal protein with sustainable plant-based options, consumers are empowered to make changes in real-time that significantly help reduce greenhouse gas emissions by choosing plant-based products over animal meat.

LegH Prep is manufactured via a genetically modified *P. pastoris* (*K. phaffii*) production strain that overexpresses LegH protein under submerged fermentation. During this process, the cells are lysed and the LegH is collected using a filtration-based recovery process. The LegH Prep contains LegH protein, host proteins, and food-grade stabilizers [4]. Health Canada [13] previously reviewed the petition to add LegH Prep to foods at a maximum soy leghemoglobin protein level of 0.8% into a variety of meat analog products and concluded that the ingredient was safe for human consumption at the intended levels of intake. The present work builds on that conclusion of safety, as no mutagenic, genotoxic, or general toxicological adverse effects due to LegH Prep administration were found in the current set of studies. The current *in vivo* studies increase the length of LegH Prep administration from 28 days [4] to 90 days, with a 28 day recovery period, reconfirming the safety of long-term ingestion of LegH Prep as demonstrated by classical *in vivo* toxicity studies conducted according to OECD protocols.

A battery of *in vivo* and *in vitro* testing has already been performed on LegH Prep to determine its safety in foods [4]. These studies have shown, under their respective testing conditions, that LegH Prep is safe to consume at the intended intake levels and does not pose any significant risk of dietary allergy or toxicity to consumers. Since 2016, LegH Prep has been incorporated into over 500 million servings of ¼ pound (113 g) meat analog products without any reported adverse effects. A new set of genotoxicity studies (bacterial reverse mutation assay and an *in vitro* Mammalian micronucleus assay in human lymphocytes) were performed to evaluate the potential of LegH Prep to induce mutations. In conclusion, based on the data collected in the mutagenicity and under the experimental conditions reported, LegH Prep derived from *P. pastoris* (*K. phaffii*) did not cause gene mutations by base pair changes or frameshifts in the genome of the five bacteria tester strains used and up to a maximum dose of 5000 *μ*g LegH/plate. Therefore, LegH Prep is nonmutagenic in this bacterial reverse mutation assay. Similarly, LegH Prep was found to be nonclastogenic/nonaneugenic in the *in vitro* mammalian micronucleus assay using human lymphocytes, which evaluated LegH Prep's potential to induce micronuclei in human lymphocytes. Precipitation of the test item in the cultures at the end of treatment was observed at 650 *μ*g/mL and higher without metabolic activation and at 250 *μ*g/mL and higher with metabolic activation in Experiment I and at 750 *μ*g/mL and higher in Experiment II. LegH Prep did not induce structural and/or numerical chromosomal damage in human lymphocytes, in agreement with the results of Fraser et al. [4]. Overall, under the conditions of this assay, the results show that LegH Prep is nonmutagenic and nonclastogenic.

Further adding to the body of work demonstrating the safety of LegH Prep as a food ingredient, a 90 day dietary study was performed in rats to evaluate the potential subchronic toxicity of LegH Prep with the addition of a 28 day recovery phase designed to follow up on any potential adverse effects observed during the 90 day study. No adverse effects were observed due to the dietary intake of LegH Prep at the maximum dose tested. The study resulted in no mortalities and no clinical observations: body weight, ophthalmological, clinical pathology, or histopathological changes due to LegH Prep administration. LegH Prep is not intended for consumption on its own but as a component of plant-based meat products. This 90 day dietary toxicity study in rats established a NOAEL of 4798.3 and 5761.5 mg/kg/day, the maximum level consumed by male and female rats, respectively. Consequently, the results of all the studies presented in this article demonstrate that the dietary consumption of LegH Prep which contains soy LegH and *P. pastoris* proteins from the production strain is not toxic under the conditions tested.

Adjusting our diets to replace animal meat with plant-based options significantly reduces the environmental impact inflicted by the animal agriculture industry. Impossible Foods' mission is to create safe food technologies that enable us to choose delicious and sustainable plant-based alternatives to animal meat, while simultaneously decreasing the environmental carbon footprint of animal agriculture.

## Figures and Tables

**Table 1:** Study design—*in vitro* mammalian micronucleus assay in HPBLs using LegH Prep.

	Without S9		With S9
	Exp. I (h)	Exp. II (h)	Exp. I (h)
Exposure period	4	44	4
Cytochalasin B exposure	40	43	40
Preparation interval	44	44	44
Total culture period^*∗*^	92	92	92

^*∗*^Exposure started 48 h after culture initiation; h = hours.

**Table 2:** Summary of bacterial reverse mutation assay results (pre-experiment I; up to 5,000 *µ*g/plate)—mean ± SD revertant colonies.

Tester strains	TA98		TA100		TA1535		TA1537		WP2 uvrA	
±S9	−S9	+S9	−S9	+S9	−S9	+S9	−S9	+S9	−S9	+S9
Water	37 ± 19.2	40 ± 3.8	104 ± 5.1	104 ± 1	13 ± 0.6	9 ± 3	11 ± 2.9	13 ± 1.7	254 ± 7	279 ± 23.6
31.6 *µ*g/plate	34 ± 2	37 ± 8.5	108 ± 4.9	105 ± 13.2	15 ± 2.1	6 ± 3.2	18 ± 1.7	13 ± 5	213 ± 36.1	242 ± 32.7
100 *µ*g/plate	33 ± 5.7	35 ± 4.9	92 ± 9.6	108 ± 7.2	9 ± 3.2	6 ± 1.2	11 ± 1.2	10 ± 1.7	228 ± 14.6	271 ± 15.6
316 *µ*g/plate	33 ± 7	37 ± 6.4	101 ± 17.1	95 ± 13.2	12 ± 1.5P	5 ± 1P	9 ± 3.1P	10 ± 2.5P	283 ± 1.85P	299 ± 18.6P
1000 *µ*g/plate	42 ± 10.5P	42 ± 6.2P	90 ± 8.7P	108 ± 7.2P	9 ± 2.9P	9 ± 4.4P	11 ± 1.2P	13 ± 3.8P	277 ± 26.9P	300 ± 26.5P
2500 *µ*g/plate	30 ± 10.4P	44 ± 8.7P	103 ± 1.7P	98 ± 7.6P	8 ± 1.2P	8 ± 1.2P	10 ± 2.5P	14 ± 5.6P	202 ± 2.6P	238 ± 30.6P
5000 *µ*g/plate	24 ± 5.8P	45 ± 2.6P	89 ± 10P	93 ± 8.7P	5 ± 3P	6 ± 0.6P	9 ± 3.1P	9 ± 4.2P	222 ± 7.6P	265 ± 22.1P
4-NOPD (10 *µ*g/plate)	542 ± 45	NA	432 ± 22	NA	918 ± 78.4	NA	60 ± 15.5	NA	1255 ± 61.2	NA
2-AA (2.5 *µ*g/plate)	NA	2057 ± 223.1	NA	1361 ± 19.2	NA	264 ± 10.1	NA	175 ± 11.9	NA	693 ± 125

2-AA = 2-aminoanthracene; LegH = soy leghemoglobin protein; NA = data not applicable; 4-NOPD = 4-nitro-o-phenylenediamine; P: visible precipitate; SD = standard deviation; +S9 = containing phenobarbital/B-naphthoflavone-induced Sprague–Dawley rat liver microsomal fraction; −S9 = without S9 microsomal fraction. Data are shown as mean ± SD revertants/plate for three replicates for each concentration in each experiment.

**Table 3:** Summary of bacterial reverse mutation assay results (pre-experiment I; up to 5,000 *µ*g/plate)—mutation factor.

Tester strains	TA98		TA100		TA1535		TA1537		WP2 uvrA	
±S9	−S9	+S9	−S9	+S9	−S9	+S9	−S9	+S9	−S9	+S9
Water	1	1	1	1	1	1	1	1	1	1
31.6 (*µ*g/plate)	0.9	0.9	1	1	1.2	0.7	1.7	1	0.8	0.9
100 (*µ*g/plate)	0.9	0.9	0.9	1	0.7	0.6	1.1	0.8	0.9	1
316 (*µ*g/plate)	0.9	0.9	1	0.9	1	0.6	0.9	0.8	1.1	1.1
1000 (*µ*g/plate)	1.1	1	0.9	1	0.7	1	1	1	1.1	1.1
2500 (*µ*g/plate)	0.8	1.1	1	0.9	0.7	0.9	1	1.1	0.8	0.9
5000 (*µ*g/plate)	0.7	1.1	0.8	0.9	0.4	0.7	0.9	0.7	0.9	0.9
4-NOPD	14.6	NA	4.1	NA	72.5	NA	5.7	NA	4.9	NA
2-AA	NA	51	NA	13.1	NA	29.3	NA	13.5	NA	2.5

2-AA = 2-aminoanthracene; LegH = soy leghemoglobin protein; NA = data not applicable; 4-NOPD = 4-nitro-o-phenylenediamine; SD = standard deviation; +S9 = containing phenobarbital/B-naphthoflavone-induced Sprague–Dawley rat liver microsomal fraction; −S9 = without S9 microsomal fraction. Data are shown as mean ± SD revertants/plate for three replicates for each concentration in each experiment.

**Table 4:** Summary of bacterial reverse mutation assay results (Experiment II; up to 5,000 *µ*g/plate)—mean ± SD revertant colonies.

Tester strains	TA98		TA100		TA1535		TA1537		WP2 uvrA	
±S9	−S9	+S9	−S9	+S9	−S9	+S9	−S9	+S9	−S9	+S9
Water	48 ± 4.4	53 ± 2.1	92 ± 13	113 ± 6.8	9 ± 4.2	12 ± 5.5	19 ± 7.8	15 ± 1.2	236 ± 25.7	345 ± 11
31.6 (*µ*g/plate)	48 ± 2.3	55 ± 5.3	123 ± 11.4	118 ± 10.2	10 ± 6.2	12 ± 3.6	18 ± 5.1	19 ± 3	196 ± 21.6	307 ± 23.7
100 (*µ*g/plate)	50 ± 7.6	46 ± 5.6	106 ± 0.6	120 ± 5.2	13 ± 4.5	14 ± 9	12 ± 3.1	19 ± 1.2	236 ± 27	256 ± 28.5
316 (*µ*g/plate)	45 ± 9.3P	37 ± 3.8P	119 ± 6.9P	107 ± 12.5P	9 ± 1.5P	13 ± 3.5P	13 ± 2P	16 ± 3.5P	265 ± 18.6P	277 ± 3.5P
1000 (*µ*g/plate)	42 ± 17.5P	55 ± 3.8P	102 ± 8.5P	112 ± 9.2P	10 ± 5.7P	13 ± 3.5P	16 ± 2P	17 ± 5.7P	229 ± 35P	300 ± 11.2P
2500 (*µ*g/plate)	46 ± 6.8P	48 ± 6.5P	106 ± 15.5P	112 ± 20.4P	9 ± 2.6P	12 ± 6.2P	10 ± 4P	20 ± 4.7P	228 ± 21.9P	285 ± 45.2P
5000 (*µ*g/plate)	52 ± 3.2P	42 ± 5.7P	122 ± 13.5P	100 ± 13.2P	9 ± 2.6P	13 ± 4.5P	8 ± 2.6P	16 ± 1P	231 ± 15.1P	337 ± 27.5P
4-NOPD (*µ*g/plate)	545 ± 52	NA	891 ± 72	NA	1570 ± 13.1	NA	96 ± 6.4	NA	1912 ± 172.1	NA
2-AA (*µ*g/plate)	NA	1771 ± 130.6	NA	1054 ± 230.3	NA	173 ± 18	NA	120 ± 11.9	NA	1177 ± 142.7

2-AA = 2-aminoanthracene; LegH = soy leghemoglobin protein; NA = data not applicable; 4-NOPD = 4-nitro-o-phenylenediamine; P: visible precipitate; SD = standard deviation; +S9 = containing phenobarbital/B-naphthoflavone-induced Sprague–Dawley rat liver microsomal fraction; −S9 = without S9 microsomal fraction. Data are shown as mean ± SD revertants/plate for three replicates for each concentration in each experiment.

**Table 5:** Summary of bacterial reverse mutation assay results of LegH Prep (Experiment II; up to 5,000 *µ*g/plate)—mutation factor.

Tester strains	TA98		TA100		TA1535		TA1537		WP2 uvrA	
±S9	−S9	+S9	−S9	+S9	−S9	+S9	−S9	+S9	−S9	+S9
Water	1	1	1	1	1	1	1	1	1	1
31.6 (*µ*g/plate)	1	1	1.3	1	1.1	1	1	1.2	0.8	0.9
100 (*µ*g/plate)	1	0.9	1.2	1.1	1.4	1.1	0.6	1.3	1	0.7
316 (*µ*g/plate)	0.9	0.7	1.3	0.9	1	1	0.7	1.1	1.1	0.8
1000 (*µ*g/plate)	0.9	1	1.1	1	1	1	0.8	1.1	1	0.9
2500 (*µ*g/plate)	1	0.9	1.2	1	0.9	1	0.5	1.3	1	0.8
5000 (*µ*g/plate)	1.1	0.8	1.3	0.9	1	1.1	0.4	1	1	1
4-NOPD	11.4	NA	9.7	NA	168.2	NA	5	NA	8.1	NA
2-AA	NA	33.6	NA	9.3	NA	14	NA	7.8	NA	3.4

2-AA = 2-aminoanthracene; LegH = soy leghemoglobin protein; NA = data not applicable; 4-NOPD = 4-nitro-o-phenylenediamine; +S9 = containing phenobarbital/B-naphthoflavone-induced Sprague–Dawley rat liver microsomal fraction; −S9 = without S9 microsomal fraction.

**Table 6:** LegH Prep concentrations used with and without metabolic (S9) activation in the *in vitro* mammalian micronucleus assay.

	−S9	+S9
Experiment I (4 hours)	162.5, 325, and 650 *µ*g/mL	2.5, 125, and 250 *µ*g/mL
Experiment II (44 hours)	250, 500, and 750 *µ*g/mL	None

**Table 7:** Summary result table for Experiments I and II, micronucleus induction in human lymphocytes, 4 h treatment, 44 h fixation interval; without metabolic activation (−S9).

	Dose group	Concentration (*µ*g/mL)	No. of cells evaluated	Cytostasis (%)	Relative cell growth (%)	Micronucleated cells frequency (%)	Historical control limits negative control	*P*	Statistical significant increase^a^
Experiment I 4 h treatment, 44 h fixation interval	*C*	0	2000	0	100	0.60	0.17%–1.17%	—	—
	2	162.5	1773	30	70	1.18		No	−
	3	325	2000	0^*∗*^	110	0.50		No	−
	4	650	2000	0^*∗*^	105	0.55		Yes	−
	MMS	65	1260	25	75	3.30		No	+
	Colc	0.4	1096	53	47	1.85		No	+

Experiment II, 44 h treatment, 44 h fixation interval	*C*	0	2000	0	100	1.05	0.17%–1.17%	—	—
	4	250	2000	25	75	1.15		No	−
	5	500	2000	24	76	0.45		No	(+)
	6	750	2000	21	79	0.45		Yes	(+)
	MMS	50	2000	13	87	2.35		No	+
	Colc	0.04	1020	71	29	4.42		No	+

*C*: negative control (culture medium); *P*: precipitation (yes: precipitation, no: no precipitation); a: statistically significant increase compared to negative control (*χ*^2^ test, *p* < 0.05). +: significant increase; (+): significant decrease; −: not significant. MMS: Methyl methanesulfonate, positive control (without metabolic activation). Colc: colchicine, positive control (without metabolic activation). CPA: cyclophosphamide, positive control (with metabolic activation). Relative cell growth: 100 × ((CBPI Test conc − 1)/(CBPI control − 1)). Cytostasis (%) = 100 − relative cell growth (%). ^*∗*^: the cytostasis is defined 0, when the relative cell growth exceeds 100%. S9: rat liver metabolic fraction.

**Table 8:** Summary result table for Experiment I, micronucleus induction in human lymphocytes, 4 h treatment, 44 h fixation interval, with metabolic activation (+S9).

	Dose group	Concentration (*µ*g/mL)	No. of cells evaluated	Cytostasis (%)	Relative cell growth (%)	Micronucleated cells' frequency (%)	Historical control limits negative control	*P*	Statistical significant increase^a^
Experiment I, 4 h treatment, 44 h fixation interval	C	0	1725	0	100	1.04	0.17%–1.17%	—	—
	2	62.5	2000	0^*∗*^	106	0.40		No	(+)
	3	125	2000	0^*∗*^	110	0.35		No	(+)
	4	250	2000	0^*∗*^	131	0.55		Yes	—
	CPA	15	1700	26	74	3.46		No	+

*C*: negative control (culture medium). *P*: precipitation (yes: precipitation, no: no precipitation); a: statistically significant increase compared to negative control (*χ*^2^ test, *p* < 0.05). +: significant increase; (+): significant decrease; −: not significant. MMS: methyl methanesulfonate, positive control (without metabolic activation). Colc: colchicine, positive control (without metabolic activation). CPA: cyclophosphamide, positive control (with metabolic activation). Relative cell growth: 100 × ((CBPI test conc − 1)/(CBPI control − 1)). Cytostasis (%) = 100 − relative cell growth (%). ^*∗*^: the cytostasis is defined 0, when the relative cell growth exceeds 100%. S9: rat liver metabolic fraction.

**Table 9:** Summary of mean body weights (g)—14 day study^*∗*^.

Day(s) relative to start date		Group 1 (0 ppm)		Group 2 (50,000 ppm)		Group 3 (100,000 ppm)		Group 4 (150,000 ppm)	
		M^*∗∗*^	F	M	F	M	F	M	F
0	Mean ± SD	217.8 ± 15.2	182.4 ± 13.6	216.6 ± 16.5	182.6 ± 14.4	218.8 ± 16.7	185.6 ± 13.1	217.2 ± 17.1	186.2 ± 11.5
3	Mean ± SD	246 ± 16.6	193.6 ± 13.4	248.2 ± 15.5	195.8 ± 19.7	245.2 ± 18	195.2 ± 15.1	240.2 ± 18.6	195.6 ± 10.7
7	Mean ± SD	283 ± 15.2	203.4 ± 16.8	282.8 ± 16.5	204.6 ± 23.3	274.2 ± 19.7	206.2 ± 14.2	266.8 ± 16.4	202.4 ± 10.7
10	Mean ± SD	305.8 ± 13.3	209.0 ± 14.3	304.2 ± 19.6	214.8 ± 25	298.8 ± 21.4	216.2 ± 20.4	288.8 ± 16	211.2 ± 13.6
14	Mean ± SD	329.2 ± 13.3	214.8 ± 17.4	328.4 ± 19.1	221.0 ± 21.9	319.8 ± 21.7	223.4 ± 20	306.8 ± 15.2	212.4 ± 15.9

^*∗*^One-way repeat ANOVA and Dunnett's test. F = female; M = male; SD = standard deviation. ^*∗∗*^*N* = 5/sex/group.

**Table 10:** Summary table of mean daily dietary intake of LegH prep-14-day study.

Day(s) relative to start date		Group 1 (0 ppm)		Group 2 (50000 ppm)		Group 3 (100,000 ppm)		Group 4 (150000 ppm)	
		M^#^	F	M	F	M	F	M	F
0 ⟶ 3	Mean ± SD	0 ± 0	0 ± 0	5085.2 ± 157.4	4451.3 ± 321.8	9507.2 ± 409.6	8529 ± 698.6	13316.9 ± 469.8	12666.9 ± 1312.3
3 ⟶ 7	Mean ± SD	0 ± 0	0 ± 0	4918.8 ± 326.5	4258 ± 275.8	9259.4 ± 490.5	9487.9 ± 2064.2	13938.6 ± 630.8	12760.6 ± 1161.2
7 ⟶ 10	Mean ± SD	0 ± 0	0 ± 0	4419.4 ± 163	4124.4 ± 228.8	8608.8 ± 509.9	8294.5 ± 510.2	12614.7 ± 573.3	12912.7 ± 1161.2
10 ⟶ 14	Mean ± SD	0 ± 0	0 ± 0	4186.3 ± 228.0	3885.8 ± 443.6	8051.9 ± 491.4	8211.1 ± 714.5	12084.3 ± 477.3	11331.0 ± 700.6
0 ⟶ 14	Mean ± SD	0 ± 0	0 ± 0	4646.9 ± 198.9	4175.9 ± 215.6	8843.5 ± 427.9	8686 ± 883.2	13035.9 ± 435.5	12401.5 ± 1208.4

F = female; M = male; SD = standard deviation. ^#^*N* = 5/sex/group.

**Table 11:** Summary of Hematology results—14 day study^a^.

Day(s) relative to start date (15)	Statistical term			Group 1 (0 ppm)		Group 2 (50,000 ppm)		Group 3 (100,000 ppm)		Group 4 (150,000 ppm)	
Parameter	M	F		M	F	M	F	M	F	M	F

ABAS (×10^3^/*µ*l)	a	a	Mean ± SD	0.040 ± 0.021	0.054 ± 0.038	0.036 ± 0.023	0.050 ± 0.016	0.032 ± 0.008	0.084 ± 0.025	0.038 ± 0.008	0.102 ± 0.078
AEOS (×10^3^/*µ*l)	a1	a1	Mean ± SD	0.062 ± 0.018	0.092 ± 0.030	0.072 ± 0.013	0.094 ± 0.051	0.088 ± 0.064	0.114 ± 0.042	0.090 ± 0.026	0.084 ± 0.042
ALUC (×10^3^/*µ*l)	a2	a1	Mean ± SD	0.074 ± 0.028	0.100 ± 0.038	0.076 ± 0.043	0.078 ± 0.029	0.070 ± 0.007	0.116 ± 0.046	0.064 ± 0.011	0.118 ± 0.028
ALYM (×10^3^/*µ*l)	a	a1	Mean ± SD	6.480 ± 1.799	7.102 ± 2.332	5.764 ± 1.347	6.132 ± 1.289	5.574 ± 0.777	8.138 ± 2.673	6.252 ± 1.066	7.478 ± 1.498
AMON (×10^3^/*µ*l)	a	a1	Mean ± SD	0.298 ± 0.113	0.224 ± 0.059	0.296 ± 0.138	0.198 ± 0.085	0.230 ± 0.078	0.316 ± 0.165	0.276 ± 0.088	0.196 ± 0.083
ANEU (×10^3^/*µ*l)	a	a	Mean ± SD	1.528 ± 0.364	0.614 ± 0.262	1.274 ± 0.214	0.600 ± 0.303	1.430 ± 0.371	0.838 ± 0.348	1.356 ± 0.337	0.690 ± 0.262
ARET (×10^3^/*µ*l)	a	a1	Mean ± SD	309.240 ± 48.018	190.220 ± 57.703	325.880 ± 41.767	180.140 ± 16.351	329.020 ± 27.211	195.500 ± 23.209	312.620 ± 43.009	195.860 ± 49.143
HCT (%)	a	a1	Mean ± SD	47.94 ± 3.30	49.02 ± 2.59	49.10 ± 2.68	51.22 ± 2.83	49.44 ± 1.90	52.64 ± 1.45	49.82 ± 2.70	53.54 ± 3.43
HGB (g/dL)	a^*∗*^	a^*∗*^	Mean ± SD	14.60 ± 0.97	15.16 ± 0.76	14.90 ± 0.67	16.10 ± 0.57	14.88 ± 0.45	16.18 ± 0.48	15.02 ± 0.82	16.42 ± 0.91
MCV (fL)	a1^*∗*^	a^*∗*^	Mean ± SD	66.30 ± 3.76	61.60 ± 1.56	65.98 ± 2.87	62.08 ± 2.64	66.38 ± 0.73	61.46 ± 1.84	66.42 ± 1.13	61.64 ± 0.97
MCH (pg)	a1^*∗*^	a^*∗*^	Mean ± SD	20.18 ± 1.33	19.02 ± 0.46	20.06 ± 0.94	19.42 ± 0.94	19.98 ± 0.26	18.88 ± 0.41	20.04 ± 0.46	18.94 ± 0.36
MCHC (g/dL)	a^*∗*^	a^*∗*^	Mean ± SD	30.46 ± 0.47	30.86 ± 0.59	30.40 ± 0.47	31.32 ± 0.67	30.08 ± 0.38	30.76 ± 0.27	30.18 ± 0.47	30.80 ± 0.20
PLT (×10^3^/*µ*l)	a^*∗*^	a^*∗*^	Mean ± SD	1020.80 ± 86.91	1127.20 ± 297.47	1012.00 ± 74.26	948.80 ± 198.92	1100.60 ± 167.04	1038.60 ± 127.40	1091.40 ± 125.43	1065.80 ± 205.45
RBC (×10^3^/*µ*l)	a^*∗*^	a^*∗*^	Mean ± SD	7.230 ± 0.378	7.986 ± 0.549	7.454 ± 0.414	8.304 ± 0.620	7.450 ± 0.239	8.574 ± 0.394	7.500 ± 0.308	8.692 ± 0.614
RDW (%)	a^*∗*^	a^*∗*^	Mean ± SD	13.34 ± 0.46	10.68 ± 0.15	13.42 ± 0.45	10.70 ± 0.28	13.44 ± 0.34	10.80 ± 0.14	13.56 ± 0.32	10.74 ± 0.27
WBC (×10^3^/*µ*l)	a^*∗*^	a^*∗*^	Mean ± SD	8.486 ± 2.235	8.180 ± 2.547	7.522 ± 1.587	7.158 ± 1.611	7.414 ± 1.217	10.092 ± 4.067	8.072 ± 1.427	8.678 ± 1.652

^a^(*N* = 5/sex/group). M: male; F: female; LegH: leghemoglobin protein; SD: standard deviation; ABAS; absolute basophil; AEOS: absolute eosinophil; ALUC: absolute large unstained cell; ALYM: absolute lymphocyte; AMON: absolute monocyte; ARET: absolute reticulocyte; ANEU: absolute neutrophil (all forms); HCT: hematocrit; HGB: hemoglobin; MCH: mean corpuscular (cell) hemoglobin; MCHC: mean corpuscular (cell) hemoglobin concentration; MCV: mean corpuscular (cell) volume; PLT: platelet count; RBC: red blood cell count; RDW: red cell distribution width; WBC: white blood cell count. Statistical terms males: a: ANOVA and Dunnett test; a1: ANOVA and Dunnett test (log); a2: ANOVA and Dunnett test (rank); a^*∗*^: ANOVA and Dunnett test; a1^*∗*^: ANOVA and Dunnett test (rank). Statistical terms females: a: ANOVA and Dunnett test (log); a1: ANOVA and Dunnett test; a^*∗*^: ANOVA and Dunnett test.

**Table 12:** Summary of clinical chemistry—14 day study (*n* = 5/sex/group).

Day(s) relative to start date (15)	Statistical term		Group 1 (0 ppm)			Group 2 (50,000 ppm)		Group 3 (100,000 ppm)		Group 4 (150,000 ppm)	
Parameter	M	F		M	F	M	F	M	F	M	F

ALT (U/L)	a	a	Mean ± SD	25.6 ± 3.1	22.8 ± 3	29.8 ± 8.3	26 ± 13.4	33.2 ± 8.8	25.2 ± 4.3	34.8 ± 9.5	56.8 ± 52.6
ALB (g/dL)	a1	a1	Mean ± SD	3.98 ± 0.11	5.12 ± 0.33	4.14 ± 0.23	5 ± 0.22	4.08 ± 0.11	5.08 ± 0.41	4.16 ± 0.25	4.90 ± 0.41
ALKP (U/L)	a2	a1	Mean ± SD	210.4 ± 48	105.2 ± 38.3	197.8 ± 25.7	99.2 ± 39.8	152.4 ± 32.1	101.4 ± 23.5	148.2 ± 41.7	119.6 ± 31.5
AST (U/L)	a1	a	Mean ± SD	86 ± 6.4	77 ± 14.2	90.6 ± 14.3	111.4 ± 70.9	98.4 ± 26.6	95.8 ± 11.7	92 ± 21.8	224.8 ± 200.7
CALC (mg/dL)	a1	a1	Mean ± SD	10.88 ± 0.33	11.82 ± 0.41	10.98 ± 0.72	11.60 ± 0.58	10.96 ± 0.42	11.46 ± 0.74	11.58 ± 0.60	11.50 ± 0.34
CL (mmol/L)	a1	a1	Mean ± SD	99.96 ± 2.34	99.84 ± 1.95	101.06 ± 1.77	99.36 ± 1.86	100 ± 1.63	98.80 ± 1.19	98.50 ± 1.90	98.64 ± 0.51
CHOL (mg/dL)	a1	a1	Mean ± SD	40.2 ± 8.8	56.8 ± 12.4	43.4 ± 11.7	46 ± 6.7	45 ± 8	41.2 ± 14.3	44.2 ± 7.4	43.6 ± 3
CREA (mg/dL)	a2	a	Mean ± SD	0.170 ± 0	0.212 ± 0.029	0.170 ± 0	0.200 ± 0.039	0.170 ± 0	0.174 ± 0.005	0.172 ± 0.004	0.198 ± 0.038
CK (U/L)	a^*∗*^	a^*∗*^	Mean ± SD	312.200 ± 151.861	178 ± 35.405	241.8 ± 55.301	201 ± 35.489	201.200 ± 30.136	168.2 ± 23.952	231.800 ± 54.302	204.2 ± 74.537
GLOB (g/dL)	a1^*∗*^	a^*∗*^	Mean ± SD	1.80 ± 0.14	1.60 ± 0.14	1.58 ± 0.11	1.62 ± 0.22	1.66 ± 0.26	1.48 ± 0.16	1.78 ± 0.15	1.74 ± 0.29
GLUC (g/dL)	a^*∗*^	a^*∗*^	Mean ± SD	109 ± 22.2	150.8 ± 31.6	106.6 ± 32.1	151.2 ± 30.7	98.2 ± 11.9	116.8 ± 37.7	167.6 ± 103.3	108.0 ± 24.9
HDL (mmol/L)	a1^*∗*^	a1^*∗*^	Mean ± SD	0.720 ± 0.148	1.2 ± 0.229	0.800 ± 0.212	0.990 ± 0.130	0.780 ± 0.164	0.866 ± 0.309	0.760 ± 0.114	0.902 ± 0.054
IPHS (mg/dL)	a1^*∗*^	a^*∗*^	Mean ± SD	10.66 ± 0.65	9.96 ± 0.83	11.20 ± 1.82	10.78 ± 1.58	12.68 ± 1.55	12.06 ± 1.37	13.74 ± 0.86^*∗∗*^	14.42 ± 1.79
LDL (mmol/L)	a1^*∗*^	a1^*∗*^	Mean ± SD	0.200 ± 0.071	0.168 ± 0.061	0.180 ± 0.084	0.130 ± 0.026	0.200 ± 0.071	0.126 ± 0.030	0.240 ± 0.055	0.170 ± 0.012
K (mmol/L)	a2^*∗*^	a^*∗*^	Mean ± SD	6.980 ± 1.084	8.008 ± 2.297	6.994 ± 1.668	9.062 ± 3.296	8.524 ± 1.207^*∗*^	11.388 ± 1.168	9.382 ± 1.028^*∗∗*^	13.354 ± 3.960^*∗*^
NA (mmol/L)	a1^*∗*^	a^*∗*^	Mean ± SD	139.20 ± 2.59	141.40 ± 3.05	141.80 ± 3.11	139.80 ± 2.77	139.60 ± 2.30	138.20 ± 2.95	139.80 ± 3.03	137 ± 2.83
SDH (U/L)	a^*∗∗*^	a^*∗∗*^	Mean ± SD	18.12 ± 9.67	16.46 ± 4.61	22.44 ± 11.80	31.90 ± 31.94	33.54 ± 20.92	34.58 ± 19	25.90 ± 11.02	76.96 ± 74.25
BILI (mg/dL)	a^*∗∗*^	a1^*∗∗*^	Mean ± SD	0.080 ± 0.019	0.088 ± 0.031	0.104 ± 0.013	0.094 ± 0.018	0.088 ± 0.019	0.110 ± 0.020	0.082 ± 0.015	0.112 ± 0.016
TP (g/dL)	a^*∗∗*^	a1^*∗∗*^	Mean ± SD	5.78 ± 0.25	6.72 ± 0.40	5.72 ± 0.31	6.62 ± 0.33	5.74 ± 0.26	6.56 ± 0.51	5.94 ± 0.27	6.64 ± 0.22
TRIG (mg/dL)	a^*∗∗*^	a1^*∗∗*^	Mean ± SD	43.2 ± 11.7	39.8 ± 5.1	55.6 ± 19.9	39.4 ± 10.2	66.8 ± 15.1	44.6 ± 7.3	65.2 ± 14.3	48.6 ± 8
BUN (mg/dL)	a1^*∗∗*^	a1^*∗∗*^	Mean ± SD	11.4 ± 3.6	12 ± 0.7	11.8 ± 1.3	13.2 ± 2.3	10.8 ± 0.4	12 ± 1.6	12.8 ± 4.2	13.4 ± 1.5

M: male; F: female; LegH: leghemoglobin protein; SD: standard deviation; ALB: albumin; ALKP: alkaline phosphatase; ALT: alanine aminotransferase; AST: aspartate aminotransferase; BUN: blood urea nitrogen; CK: creatinine phosphokinase; CALC: calcium; CHOL: cholesterol; CL: chloride; CREA: creatinine; ELISA: enzyme-linked immunosorbent assay; GLOB: globulin; GGT: gamma-glutamyl transferase; GLUC: glucose; HDL: high density lipoprotein cholesterol; K: potassium; LDL: low density lipoprotein cholesterol; Na: sodium; IPHS: inorganic phosphorous; SDH: sorbitol dehydrogenase; T3: triiodothyronine; T4: thyroxine; BILI: total bilirubin; TP: total protein; TRIG: triglycerides; TSH: thyroid-stimulating hormone; U/L: units/liter. Statistical terms males: a: ANOVA and Dunnett test (log); a1: ANOVA and Dunnett test; a2: ANOVA and Dunnett test (rank); a^*∗*^: ANOVA and Dunnett test (log); a1^*∗*^: ANOVA and Dunnett test; ^*∗∗*^ = *p* < 0.01; a2^*∗*^: ANOVA and Dunnett test (rank)^*∗*^ = *p* < 0.05 = *p* < 0.01; a^*∗∗*^: ANOVA and Dunnett test; a1^*∗∗*^: ANOVA and Dunnett test (rank). Statistical terms females: a: ANOVA and Dunnett test (rank); a1: ANOVA and Dunnett test; a^*∗*^: ANOVA and Dunnett test (rank); ^*∗*^ = *p* < 0.05; ^*∗∗∗*^ = *p* < 0.01; a1^*∗*^: ANOVA and Dunnett test (rank); a^*∗∗*^: ANOVA and Dunnett test (log); a1^*∗∗*^: ANOVA and Dunnett test.

**Table 13:** Summary of mean body weights—90 day dietary study—main phase^a,^^*∗*^.

Day(s) relative to start date		Group 1 (0 ppm)		Group 2 (30,000 ppm)		Group 3 (60,000 ppm)		Group 4 (90,000 ppm)	
		M	F	M	F	M	F	M	F
0	Mean ± SD	241.3 ± 14.9	166.3 ± 12.1	242.6 ± 16.6	166.4 ± 12.0	242.9 ± 17.8	167.3 ± 11.6	244.1 ± 17.4	166.6 ± 12.4
	N	15	15	10	10	10	10	15	15

7	Mean ± SD	302.5 ± 15.3	199.1 ± 14.9	306.9 ± 18.1	201.8 ± 17.1	309.8 ± 22.1	196.6 ± 13.0	304.1 ± 19.2	194.1 ± 15.7
	N	15	15	10	10	10	10	15	15

14	Mean ± SD	352.1 ± 16.6	226.2 ± 21.6	357.7 ± 20.4	228.5 ± 18.6	360.5 ± 27.2	223.2 ± 16.0	353.3 ± 24.5	220.6 ± 19.7
	N	15	15	10	10	10	10	15	15

21	Mean ± SD	393.5 ± 18.7	247.6 ± 26.4	399.4 ± 23.1	246.6 ± 20.7	403.1 ± 34.5	242.0 ± 16.8	392.5 ± 26.5	242.3 ± 21.8
	N	15	15	10	10	10	10	15	15

27	Mean ± SD	427.0 ± 23.3	264.7 ± 28.8	430.6 ± 30.3	264.7 ± 26.0	437.0 ± 40.0	256.5 ± 14.3	426.3 ± 27.5	256.4 ± 24.7
	N	15	15	10	10	10	10	15	15

34	Mean ± SD	456.4 ± 25.8	274.1 ± 31.5	456.5 ± 33.5	277.7 ± 24.2	467.3 ± 42.9	270.0 ± 17.2	457.7 ± 28.1	272.3 ± 27.3
	N	15	15	10	10	10	10	15	15

41	Mean ± SD	488.1 ± 29.1	288.2 ± 30.6	484.3 ± 37.9	288.1 ± 25.5	497.4 ± 47.4	282.5 ± 16.4	484.8 ± 28.7	286.1 ± 28.1
	N	15	15	10	10	10	10	15	15

49	Mean ± SD	522.7 ± 34.1	298.0 ± 35.5	516.4 ± 41.9	300.4 ± 29.5	530.3 ± 50.3	294.8 ± 21.1	515.9 ± 30.9	298.7 ± 32.0
	N	15	15	10	10	10	10	15	15

56	Mean ± SD	543.9 ± 36.7	301.7 ± 38.6	537.5 ± 43.7	308.2 ± 31.5	550.1 ± 53.3	300.8 ± 20.8	534.7 ± 33.4	305.6 ± 34.7
	N	15	15	10	10	10	10	15	15

63	Mean ± SD	559.6 ± 36.5	309.0 ± 38.7	550.5 ± 44.3	312.0 ± 32.3	563.0 ± 54.6	306.7 ± 20.4	550.5 ± 37.6	313.3 ± 33.2
	N	15	15	10	10	10	10	15	15

70	Mean ± SD	577.0 ± 37.7	316.1 ± 38.2	566.3 ± 47.2	319.3 ± 32.1	578.9 ± 55.6	313.4 ± 20.1	567.3 ± 39.6	320.5 ± 35.2
	N	15	15	10	10	10	10	15	15

77	Mean ± SD	592.5 ± 40.6	327.8 ± 38.2	582.4 ± 49.6	329.2 ± 33.9	591.8 ± 54.9	322.3 ± 24.8	583.0 ± 42.4	328.7 ± 38.3
	N	15	15	10	10	10	10	15	15

84	Mean ± SD	605.4 ± 40.6	334.5 ± 41.2	596.6 ± 50.6	330.9 ± 31.5	603.1 ± 57.5	326.6 ± 26.4	597.5 ± 45.0	330.9 ± 39.4
	N	15	15	10	10	10	10	15	15

91	Mean ± SD	616.5 ± 42.6	338.9 ± 44.6	607.5 ± 53.1	336.3 ± 36.9	614.1 ± 60.7	330.3 ± 28.4	611.9 ± 48.3	335.4 ± 40.5
	N	15	15	10	10	10	10	15	15

^a^One-way repeat ANOVA and Dunnett test. ^*∗*^*N* = 10/sex/group. M: male; F: female; LegH: leghemoglobin protein; SD: standard deviation.

**Table 14:** Summary of mean body weights in 90 day study (g) recovery phase^a,^^*∗*^.

LegH dose levels (study day)		Group 1 (0 ppm)		Group 4 (90,000 ppm)	
		M	F	M	F
91	Mean ± SD	644.4 ± 54.1	338.4 ± 30.8	624.6 ± 62.9	322 ± 27.6
98	Mean ± SD	656.8 ± 54	346.6 ± 32.1	637.6 ± 62.6	332.8 ± 29.6
105	Mean ± SD	665.6 ± 56.2	351.6 ± 34.2	645.2 ± 61.0	335.6 ± 29
112	Mean ± SD	678.8 ± 55.3	358.8 ± 32.9	656.8 ± 61.6	343.6 ± 32.7
119	Mean ± SD	688.6 ± 55.9	367.8 ± 41	668.4 ± 61.9	352.2 ± 39.3

^a^One-way repeat ANOVA and Dunnett test. ^*∗*^*N* = 5/sex/group. M: male; F: female; LegH: leghemoglobin protein; SD: standard deviation.

**Table 15:** Summary of mean daily dietary intake of LegH Prep (mg/kg/day)—90 day dietary study^a,^^*∗*^.

Day(s) relative to start date		Group 1 (0 ppm)		Group 2 (30,000 ppm)		Group 3 (60,000 ppm)		Group 4 (90,000 ppm)	
		M	F	M	F	M	F	M	F
0 ⟶ 3	Mean ± SD	0	0	3000.5 ± 207.6	3174.5 ± 357.5	6008.2 ± 435.3	6095.1 ± 604.2	8723.2 ± 642.1	9007.0 ± 705.3
	*N*	15	15	10	10	10	10	15	15

3 ⟶ 7	Mean ± SD	0	0	2458.5 ± 152.8	2664.2 ± 120.6	4994.0 ± 346.8	5629.4 ± 809.1	7479.9 ± 458.6	7949.8 ± 415.5
	*N*	15	15	10	10	10	10	15	15

10 ⟶ 14	Mean ± SD	0	0	2101.0 ± 119.7	2548.3 ± 328.5	4287.2 ± 195.7	5023.9 ± 701.4	6420.2 ± 347.5	7460.4 ± 606.5
	*N*	15	15	10	10	10	10	15	15

14 ⟶ 17	Mean ± SD	0	0	2102.7 ± 141.1	2604.7 ± 402.4	4282.2 ± 218.2	4999.9 ± 669.1	6422.8 ± 444.4	7409.2 ± 933.4
	*N*	15	15	10	10	10	10	15	15

17 ⟶ 21	Mean ± SD	0	0	1958.1 ± 96.8	2373.7 ± 220.4	3909.5 ± 217.9	4762.2 ± 439.7	5891.9 ± 355.1	7004.4 ± 629.1
	*N*	15	15	10	10	10	10	15	15

21 ⟶ 24	Mean ± SD	0	0	1900.2 ± 148.1	2336.8 ± 524.3	3867.7 ± 202.2	4747.6 ± 727.2	5817.9 ± 301.1	6949.8 ± 1268.4
	*N*	15	15	10	10	10	10	15	15

24 ⟶ 27	Mean ± SD	0	0	1822.8 ± 110.1	2245.0 ± 251.9	3622.9 ± 186.5	4714.0 ± 543.6	5516.8 ± 316.1	6781.0 ± 589.8
	*N*	15	15	10	10	10	10	15	15

27 ⟶ 30	Mean ± SD	0	0	1720.3 ± 173.2	2155.3 ± 327.7	3621.3 ± 210.6	4598.0 ± 874.2	5678.8 ± 350.1	6800.8 ± 789.4
	*N*	15	15	10	10	10	10	15	15

30 ⟶ 34	Mean ± SD	0	0	1816.3 ± 120.1	2229.1 ± 363.5	3550.9 ± 202.9	4407.7 ± 407.8	5382.0 ± 321.8	6535.6 ± 799.2
	*N*	15	15	10	10	10	10	15	15

34 ⟶ 37	Mean ± SD	0	0	1725.1 ± 116.9	2018.9 ± 354.9	3469.2 ± 157.1	4539.6 ± 1069.3	5159.3 ± 329.2	6062.9 ± 1044.3
	*N*	15	15	10	10	10	10	15	15

37 ⟶ 41	Mean ± SD	0	0	1712.2 ± 115.5	2201.2 ± 405.9	3402.0 ± 137.8	4482.4 ± 769.6	5156.4 ± 313.4	6382.9 ± 497.8
	*N*	15	15	10	10	10	10	15	15

41 ⟶ 45	Mean ± SD	0	0	1617.6 ± 117.8	2021.5 ± 280.4	3234.8 ± 97.6	4247.5 ± 1025.7	4857.5 ± 271.5	5832.4 ± 608.1
	*N*	15	15	10	10	10	10	15	15

45 ⟶ 49	Mean ± SD	0	0	1566.6 ± 95.4	1878.7 ± 283.0	3055.0 ± 99.8	3856.7 ± 342.8	4599.8 ± 263.0	5566.2 ± 635.4
	*N*	15	15	10	10	10	10	15	15

49 ⟶ 52	Mean ± SD	0	0	1539.3 ± 77.0	1797.6 ± 447.1	2995.0 ± 101.7	3792.3 ± 643.0	4529.9 ± 240.7	5462.4 ± 558.8
	*N*	15	15	10	10	10	10	15	15

52 ⟶ 56	Mean ± SD	0	0	1492.2 ± 107.4	1913.8 ± 302.1	2833.2 ± 133.8	3794.1 ± 641.8	4286.4 ± 337.6	5380.4 ± 498.0
	*N*	15	15	10	10	10	10	15	15

56 ⟶ 59	Mean ± SD	0	0	1387.5 ± 93.1	1695.4 ± 246.4	2673.8 ± 147.6	3594.1 ± 705.2	4095.9 ± 279.9	5171.3 ± 541.3
	*N*	15	15	10	10	10	10	15	15

59 ⟶ 63	Mean ± SD	0	0	1374.8 ± 84.8	1760.8 ± 302.5	2817.4 ± 427.5	3616.0 ± 349.9	4028.4 ± 170.3	5070.7 ± 506.4
	*N*	15	15	10	10	10	10	15	15

63 ⟶ 66	Mean ± SD	0	0	1467.3 ± 245.9	1614.7 ± 343.6	2683.7 ± 141.6	3400.2 ± 349.1	4057.6 ± 312.0	5066.8 ± 703.4
	*N*	15	15	10	10	10	10	15	15

66 ⟶ 70	Mean ± SD	0	0	1388.4 ± 154.4	1784.4 ± 285.6	2596.6 ± 164.3	3437.1 ± 355.7	3943.3 ± 231.7	5048.8 ± 561.0
	*N*	15	15	10	10	10	10	15	15

70 ⟶ 73	Mean ± SD	0	0	1367.8 ± 72.2	1738.1 ± 373.6	2659.4 ± 285.4	3585.6 ± 740.3	3903.6 ± 206.1	4876.1 ± 547.1
	*N*	15	15	10	10	10	10	15	15

73 ⟶ 77	Mean ± SD	0	0	1370.9 ± 146.5	1826.3 ± 325.0	2617.1 ± 154.2	3538.9 ± 440.3	3884.5 ± 201.2	4950.3 ± 426.3
	*N*	15	15	10	10	10	10	15	15

77 ⟶ 80	Mean ± SD	0	0	1319.2 ± 141.4	1792.9 ± 490.7	2361.8 ± 164.4	3101.6 ± 385.3	3589.5 ± 190.5	4815.3 ± 674.4
	*N*	15	15	10	10	10	10	15	15

80 ⟶ 84	Mean ± SD	0	0	1282.1 ± 91.4	1565.5 ± 177.1	2322.9 ± 110.6	3336.5 ± 331.9	3576.2 ± 262.8	4759.6 ± 633.3
	N	15	15	10	10	10	10	15	15

84 ⟶ 87	Mean ± SD	0	0	1244.6 ± 128.8	1658.0 ± 189.7	2490.8 ± 160.8	3398.7 ± 608.1	3689.1 ± 378.3	4909.4 ± 797.1
	*N*	15	15	10	10	10	10	15	15

87 ⟶ 91	Mean ± SD	0	0	1217.5 ± 125.1	1525.5 ± 122.6	2263.5 ± 79.5	3272.0 ± 325.3	3513.1 ± 288.2	4788.4 ± 656.3
	*N*	15	15	10	10	10	10	15	15

0 ⟶ 91	Mean ± SD	0	0	1637.3 ± 63.2	2024.8 ± 227.5	3202.3 ± 83.4	4127.9 ± 437.1	4820.4 ± 176.9	5930.8 ± 398.2
	*N*	15	15	10	10	10	10	15	15

^a^One-way repeat ANOVA and Dunnett test. ^*∗*^*N* = 10/sex/group except for recovery groups = 15/sex/group. M: male; F: female; LegH: leghemoglobin protein; SD: standard deviation.

**Table 16:** Summary of mean terminal body weights and organ weights (g)—90 day dietary study.

LegH dose levels			Group 1 (0 ppm)			Group 2 (30,000 ppm)		Group 3 (60,000 ppm)		Group 4 (90,000 ppm)	
Parameter	Statistical term			M	F	M	F	M	F	M	F

Terminal BW	M	F	Mean ± SD	583.8 ± 27.7	326.5 ± 51.4	586.5 ± 52.1	321.8 ± 34.6	591.6 ± 60	316.1 ± 27.7	585.2 ± 40.7	330.5 ± 44.1
Adrenal	a	d	Mean ± SD	0.0594 ± 0.0169	0.0653 ± 0.0096	0.587 ± 0.0144	0.0642 ± 0.0128	0.0618 ± 0.0174	0.0729 ± 0.0114	0.0522 ± 0.0121	0.0705 ± 0.011
Brain	a	d	Mean ± SD	2.297 ± 0.049	2.11 ± 0.112	2.201 ± 0.114	2.069 ± 0.093	2.258 ± 0.09	2.067 ± 0.076	2.264 ± 0.112	2.103 ± 0.065
Epididymis	b	d	Mean ± SD	1.5286 ± 0.0995	—	1.5826 ± 0.0703	—	1.6108 ± 0.1868	—	1.502 ± 0.1743	—
Heart	a	d	Mean ± SD	1.64 ± 0.13	1.603 ± 0.115	1.592 ± 0.162	1.008 ± 0.108	1.601 ± 0.157	1.035 ± 0.102	1.56 ± 0.132	1.088 ± 0.116
Kidneys	a	d	Mean ± SD	3.389 ± 0.334	2.185 ± 0.325	3.717 ± 0.472	2.061 ± 0.214	3.697 ± 0.298	2.119 ± 0.198	3.52 ± 0.228	2.306 ± 0.242
Liver	a	d	Mean ± SD	13.898 ± 1.404	8.582 ± 1.398	14.661 ± 2.283	7.809 ± 0.817	14.872 ± 2.082	7.986 ± 1.491	13.467 ± 1.586	8.685 ± 0.906
Pituitary	a	c	Mean ± SD	0.016 ± 0.0026	0.0297 ± 0.0107	0.0798 ± 0.0047	0.0297 ± 0.0107	0.0163 ± 0.0052	0.0286 ± 0.0112	0.0183 ± 0.0056	0.0265 ± 0.0078
Pro, SV, and CG (combined)	a	—	Mean ± SD	3.36 ± 0.479	—	3.394 ± 0.436	—	3.18 ± 0.291	—	3.541 ± 0.428	—
Spleen	a	d	Mean ± SD	0.913 ± 0.081	0.584 ± 0.094	0.913 ± 0.092	0.496 ± 0.071	0.41 ± 0.125	0.521 ± 0.071	0.833 ± 0.154	0.535 ± 0.06
Testes	b	—	Mean ± SD	3.578 ± 0.308	—	3.64 ± 0.228	—	3.659 ± 0.321	—	3.672 ± 0.304	—
Thymus	c	d	Mean ± SD	0.3442 ± 0.094	0.3381 ± 0.0991	0.263 ± 0.0512^*∗*^	0.2711 ± 0.0757	0.3226 ± 0.0677	0.2316 ± 0.045^*∗∗*^	0.2586 ± 0.0596^*∗*^	0.2604 ± 0.0645
Thyroid-parathyroid	a	b	Mean ± SD	0.0261 ± 0.0076	0.0239 ± 0.0079	0.0324 ± 0.0101	0.0239 ± 0.0079	0.0309 ± 0.0065	0.0269 ± 0.002	0.0646 ± 0.0054	0.032 ± 0.052
Ovaries with oviducts	—	d	Mean ± SD	—	0.1155 ± 0.0187	—	0.1088 ± 0.0252	—	0.982 ± 0.0237	—	0.1211 ± 0.0221
Uterus	—	b	Mean ± SD	—	0.719 ± 0.187	—	0.687 ± 0.312	—	0.723 ± 0.202	—	0.657 ± 0.223

CG: coagulating gland of the prostate; M: male; F: female; LegH: leghemoglobin protein; Pro: prostate; SD: standard deviation; SV: seminal vesicle. Statistical terms: a: ANOVA and Dunnett test; b: Kruskal–Wallis and Dunn test; c: ANOVA and Dunnett test (log); ^*∗*^ = *p* < 0.05; d: ANOVA and Dunnett test; ^*∗∗*^ = *p* < 0.01; d: ANOVA and Dunnett test; ^*∗*^ = *p* < 0.05.

**Table 17:** Summary of mean terminal body weights and organ weights (g)—90 day dietary study—recovery phase.

LegH dose levels				Group 1 (0 ppm)		Group 4 (90,000 ppm)	
Parameter	Statistical term			M^#^	F	M	F

Terminal BW	M	F	Mean ± SD	665.6 ± 54.6	351.8 ± 37.3	648.2 ± 58.6	336.8 ± 36.3
Adrenal	a	d	Mean ± SD	0.0484 ± 0.0167	0.0696 ± 0.0117	0.0594 ± 0.0105	0.0698 ± 0.0193
Brain	a	d	Mean ± SD	2.33 ± 0.146	2.0998 ± 0.146	2.264 ± 0.057	2.044 ± 0.111
Epididymis	b	d	Mean ± SD	1.5596 ± 0.2282	—	1.822 ± 0.255	—
Heart	a	d	Mean ± SD	1.738 ± 0.225	1.114 ± 0.101	1.668 ± 0.092	1.014 ± 0.082
Kidneys	a	d	Mean ± SD	3.578 ± 0.0207	2.2 ± 0.199	2.932 ± 0.592	2.026 ± 0.164
Liver	a	d	Mean ± SD	15.138 ± 2.39	8.954 ± 1.223	15.736 ± 2.153	7.932 ± 1.056
Pituitary	a	c	Mean ± SD	0.0230 + 0.0067	0.025 ± 0.0072	0.0166 + 0.0025	0.0236 ± 0.0064
Pro, SV, and CG (combined)	a	—	Mean ± SD	3.616 ± 0.181	—	3.706 ± 0.861	—
Spleen	a	d	Mean ± SD	0.95 ± 0.182	0.54 ± 0.061	0.912 ± 0.038	0.56 ± 0.067
Testes	b	—	Mean ± SD	3.816 ± 0.505	—	4.088 ± 0.543	—
Thymus	c	d	Mean ± SD	0.2264 ± 0.0636	0.2354 ± 0.0289	0.196 ± 0.0701	0.2006 ± 0.0442
Thyroid-parathyroid	a	b	Mean ± SD	0.0396 ± 0.0099	0.0398 ± 0.0055	0.0444 ± 0.0081	0.034 ± 0.0052
Ovaries with oviducts	—	d	Mean ± SD	—	0.1002 ± 0.0188	—	0.1336 ± 0.0168^*∗*^
Uterus	—	b	Mean ± SD	—	0.764 ± 0.082	—	0.646 ± 0.192

^#^ *N* = 5/sex/group. CG: coagulating gland; M: male; F: female; LegH: leghemoglobin protein; Pro: prostate; SD: standard deviation; SV: seminal vesicle. Terms: a: ANOVA and Dunnett test; b: Kruskal–Wallis and Dunn; c: ANOVA and Dunnett test (log); ^*∗*^ = *p* < 0.05; d: ANOVA and Dunnett test; ^*∗∗*^ = *p* < 0.01.

**Table 18:** Hematology and coagulation—90 day dietary study.

Day(s) relative to start date (92)	Statistical term			Group 1 (0 ppm)		Group 2 (30,000 ppm)		Group 3 (60,000 ppm)		Group 4 (90,000 ppm)	
Parameter	M	F		M^*∗*^	F	M	F	M	F	M	F

ABAS (×10^3^/*µ*l)	f	a	Mean ± SD	0.128 ± 0.057	0.071 ± 0.04	0.112 ± 0.071	0.083 ± 0.038	0.096 ± 0.044	0.061 ± 0.046	0.131 ± 0.05	0.051 ± 0.019
AEOS (×10^3^/*µ*l)	a	a	Mean ± SD	0.189 ± 0.088	0.112 ± 0.06	0.191 ± 0.068	0.145 ± 0.052	0.17 ± 0.071	0.131 ± 0.051	0.171 ± 0.07	0.114 ± 0.059
ALUC (×10^3^/*µ*l)	a	a	Mean ± SD	0.06 ± 0.015	0.061 ± 0.024	0.053 ± 0.026	0.065 ± 0.032	0.076 ± 0.02	0.041 ± 0.018	0.055 ± 0.026	0.038 ± 0.016
ALYM (×10^3^/*µ*l)	g	g	Mean ± SD	7.812 ± 1.624	4.938 ± 1.889	7.241 ± 2.334	4.819 ± 1.237	7.548 ± 2.311	4.166 ± 1.03	7.47 ± 2.266	3.9 ± 0.603
AMON (×10^3^/*µ*l)	f	a	Mean ± SD	0.357 ± 0.093	0.248 ± 0.145	0.312 ± 0.132	0.273 ± 0.071	0.432 ± 0.24	0.171 ± 0.074	0.31 ± 0.13	0.172 ± 0.07
ANEU (×10^3^/*µ*l)	g	g	Mean ± SD	1.517 ± 0.631	0.989 ± 0.598	1.546 ± 0.503	0.958 ± 0.324	2.252 ± 2.161	0.854 ± 0.245	1.424 ± 0.342	1.131 ± 0.48
ARET (×10^3^/*µ*l)	f	a	Mean ± SD	186.73 ± 44.046	167.23 ± 40.967	168.52 ± 31.949	141.02 ± 31.649	177.93 ± 39.972	148.76 ± 32.58	179.76 ± 24.208	161.7 ± 37.028
HCT (%)	a	g	Mean ± SD	47.2 ± 2.16	43.99 ± 5.53	47.96 ± 1.37	45.67 ± 1.15	47.54 ± 1.65	46.4 ± 1.74	48.25 ± 1.3	46.43 ± 1.91
HGB (g/dL)	f	g	Mean ± SD	15.57 ± 0.69	14.84 ± 1.81	16.01 ± 0.28	15.37 ± 0.25	15.7 ± 0.6	15.76 ± 0.63	15.91 ± 0.38	15.71 ± 0.66
MCV (fL)	a	a	Mean ± SD	54.06 ± 2.27	54.39 ± 1.47	54.72 ± 1.31	54.7 ± 1.18	55.09 ± 2.05	54.62 ± 0.79	56.99 ± 1.94	55.68 ± 1.05
MCH (pg)	a	a	Mean ± SD	17.88 ± 0.69	18.36 ± 0.44	18.29 ± 0.34	18.44 ± 0.4	18.19 ± 0.73	18.54 ± 0.37	18.79 ± 0.61	18.83 ± 0.35
MCHC (g/dL)	a	g	Mean ± SD	33.06 ± 0.43	33.76 ± 0.36	33.42 ± 0.52	33.7 ± 0.63	33 ± 0.29	33.96 ± 0.25	32.97 ± 0.31	33.81 ± 0.21
PLT (×10^3^/*µ*l)	a	g	Mean ± SD	1071.9 ± 122.9	917.6 ± 334.34	1074.8 ± 118.09	1016.3 ± 145.61	1070.5 ± 153.07	958 ± 114.32	1050.6 ± 90.98	923 ± 230.41
RBC (×10^3^/*µ*l)	a	g	Mean ± SD	8.717 ± 0.432	8.09 ± 1.018	8.769 ± 0.21	8.349 ± 0.204	8.633 ± 0.316	8.496 ± 0.323	8.47 ± 0.238	8.343 ± 0.385
RDW (%)	g	a	Mean ± SD	13.44 ± 0.85	0.48 ± 10	13.13 ± 0.49	12.05 ± 0.5	13.56 ± 0.8	11.96 ± 0.48	13.26 ± 0.38	12.17 ± 0.31
WBC (×10^3^/*µ*l)	f	g	Mean ± SD	10.06 ± 2.258	6.42 ± 2.37	9.464 ± 2.813	6.344 ± 1.359	10.573 ± 3.491	5.423 ± 1.213	9.561 ± 2.479	5.406 ± 0.832
APTT (seconds)	a	g	Mean ± SD	15.67 ± 0.72	14.94 ± 1.87	15.39 ± 1.28	14.01 ± 1.65	17.03 ± 1.62	15.64 ± 4.09	15.89 ± 1.75	14.33 ± 1.8
PT (seconds)	a	a	Mean ± SD	9.76 ± 0.38	9.27 ± 0.22	9.98 ± 0.32	9.19 ± 0.16	10.21 ± 0.52	9.34 ± 0.2	9.88 ± 0.25	9.32 ± 0.16

^a^(*N* = 10/sex/group). M: male; F: female; LegH: leghemoglobin protein; SD: standard deviation; ABAS: absolute basophil; AEOS: absolute eosinophil; ALUC: absolute large unstained cell; ALYM: absolute lymphocyte; AMON: absolute monocyte; ARET: absolute reticulocyte; ANEU: absolute neutrophil (all forms); HCT: hematocrit; HGB: hemoglobin; MCH: mean corpuscular (cell) hemoglobin; MCHC: mean corpuscular (cell) hemoglobin concentration; MCV: mean corpuscular (cell) volume; PLT: platelet count; RBC: red blood cell count; RDW: red cell distribution width; WBC: white blood cell count; APTT: activated partial thromboplastin time; PT: prothrombin time. Statistical terms: a: ANOVA and Dunnett test; f, ANOVA and Dunnett test (log); g: ANOVA and Dunnett test (rank).

**Table 19:** Summary of clinical chemistry parameters—90 day dietary study^#^.

Day(s) relative to start date M (92), F (93)	Statistical term			Group 1 (0 ppm)		Group 2 (30,000 ppm)		Group 3 (60,000 ppm)		Group 4 (90,000 ppm)	
Parameter	M	F		M^*∗*^	F	M^#^	F	M	F	M	F

ALT (U/L)	a	a	Mean ± SD	25.4 ± 6.2	21.2 ± 9	27.3 ± 4.4	17.2 ± 3.9	42.3 ± 51.9	16.4 ± 3.2	32.6 ± 24.7	19.3 ± 4.3
ALB (g/dL)	a1	a1	Mean ± SD	4.19 ± 0.24	5.39 ± 0.65	4.31 ± 0.15	5.31 ± 0.5	4.33 ± 0.3	5.13 ± 0.55	4.38 ± 0.28	5.47 ± 0.37
ALKP (U/L)	a1	a2	Mean ± SD	67.3 ± 9.2	29.1 ± 8.7	67.6 ± 10.3	30.6 ± 9.6	66.4 ± 11.3	36 ± 9.8	59 ± 16.1	29.6 ± 5.4
AST (U/L)	a	a1	Mean ± SD	77.8 ± 17.1	71.9 ± 15.9	75.2 ± 6.9	67.8 ± 18.4	88.3 ± 48.6	68.6 ± 15.4	85.7 ± 30.1	70.2 ± 10.2
CALC (mg/dL)	a1	a1	Mean ± SD	11.47 ± 1.09	12.41 ± 1.13	11.44 ± 0.47	12.23 ± 0.92	11.68 ± 0.58	11.88 ± 1.11	11.57 ± 0.67	12.17 ± 0.57
CL (mmol/L)	a1	a1	Mean ± SD	101.57 ± 1.55	104.6 ± 1.88	101.63 ± 1.7	105.83 ± 1.49	100.43 ± 1.57	104.6 ± 2.05	101.98 ± 1.71	103.75 ± 2.35
CHOL (mg/dL)	a1	a1	Mean ± SD	63.4 ± 15.4	80.2 ± 17.7	65.7 ± 16.5	79.7 ± 14.7	78.7 ± 20.8	64.3 ± 26.9	72.6 ± 16.5	70.2 ± 18.3
CK (U/L)	a	a	Mean ± SD	212.2 ± 173.914	153.4 ± 51.728	145.556 ± 44.136	209.2 ± 200.261	151.7 ± 50.197	188 ± 116.466	230.1 ± 167.781	182.6 ± 77.879
CREA (mg/dL)	a	a3	Mean ± SD	0.276 ± 0.038	0.304 ± 0.053	0.259 ± 0.031	0.33 ± 0.071	0.265 ± 0.049	0.317 ± 0.062	0.242 ± 0.03	0.273 ± 0.047
GLOB (g/dL)	a	a3	Mean ± SD	2.38 ± 0.19	2.07 ± 0.24	2.5 ± 0.14	2.07 ± 0.19	2.33 ± 0.23	2.02 ± 0.25	2.38 ± 0.13	2.1 ± 0.21
GLUC (g/dL)	a1	a3	Mean ± SD	243.1 ± 45	208.5 ± 23.9	232.8 ± 35.3	200.2 ± 37	299.8 ± 76	216.8 ± 41.9	211.9 ± 33.3	223.7 ± 32.6
HDL (mmol/L)	a	a3	Mean ± SD	0.98 ± 0.23	1.587 ± 0.345	1.056 ± 0.27	1.594 ± 0.258	1.27 ± 0.313	1.296 ± 0.498	1.15 ± 0.31	1.43 ± 0.333
IPHS (mg/dL)	a	a3	Mean ± SD	8.47 ± 1.18	8.21 ± 1.31	9.3 ± 0.93	8.76 ± 0.78	9.62 ± 1.4	8.87 ± 1.59	9.13 ± 1.55	9.36 ± 1.38
LDL (mmol/L)	a	a4	Mean ± SD	0.21 ± 0.074	0.159 ± 0.057	0.267 ± 0.122	0.159 ± 0.053	0.35^*∗*^±0.127	0.121 ± 0.033	0.25 ± 0.071	0.141 ± 0.058
K (mmol/L)	a1	a5	Mean ± SD	7.166 ± 1.441	6.533 ± 1.403	8.223 ± 1.652	6.852 ± 1.425	8.693 ± 2.121	7.494 ± 1.435	8.078 ± 2.585	8.173 ± 2.302
NA (mmol/L)	a	a3	Mean ± SD	141.4 ± 3.03	142.3 ± 1.77	141.33 ± 1.41	143.4 ± 2.01	140.6 ± 1.9	141.6 ± 2.01	141.5 ± 2.27	141.7 ± 2.91
SDH (U/L)	a2	a1	Mean ± SD	15.52 ± 4.33	15.39 ± 5.1	19.33 ± 7.29	11.13 ± 2.26	24.9 ± 23.23	12.33 ± 5.12	23.59 ± 28.37	13.33 ± 3.96
BILI (mg/dL)	a3	a1	Mean ± SD	0.054 ± 0.018	0.075 ± 0.034	0.068 ± 0.031	0.072 ± 0.025	0.072 ± 0.023	0.064 ± 0.025	0.078 ± 0.024	0.066 ± 0.016
TP (g/dL)	a3	a1	Mean ± SD	6.57 ± 0.36	7.46 ± 0.75	6.81 ± 0.2	7.38 ± 0.52	6.66 ± 0.4	7.15 ± 0.67	6.76 ± 0.33	7.57 ± 0.48
TRIG (mg/dL)	a3	a2	Mean ± SD	85.3 ± 37	75.3 ± 42.6	82.2 ± 31.6	57 ± 24.8	99.8 ± 33.9	65.4 ± 41.1	99.2 ± 45.2	76.5 ± 38.2
BUN (mg/dL)	a3	a4	Mean ± SD	13.1 ± 1.7	17.6 ± 3.4	13.1 ± 1.5	18 ± 2.1	14 ± 1.4	16.4 ± 1.6	13.4 ± 1.4	16.8 ± 1.9

M: male; F: female; LegH: leghemoglobin protein; SD: standard deviation; ALB: albumin; ALKP: alkaline phosphatase; ALT: alanine aminotransferase; AST: aspartate aminotransferase; BUN: blood urea nitrogen; CK: creatinine phosphokinase; CALC: calcium; CHOL: cholesterol; CL: chloride; CREA: creatinine; ELISA: enzyme-linked immunosorbent assay; GLOB: globulin; GGT: gamma-glutamyl transferase; GLUC: glucose; HDL: high density lipoprotein cholesterol; K: potassium; LDL: low density lipoprotein cholesterol; NA: sodium; IPHS: inorganic phosphorous; SDH: sorbitol dehydrogenase; T3: triiodothyronine; T4: thyroxine; BILI: total bilirubin; TP: total protein; TRIG: triglycerides; TSH: thyroid-stimulating hormone; U/L: units/liter. Statistical Terms males: a: ANOVA and Dunnett test; ^*∗*^ = *p* < 0.05; a1: Anova and Dunnett test (log); a2: ANOVA and Dunnett test (rank); a3: ANOVA and Dunnett test. Statistical terms females: a: ANOVA and Dunnett test (rank), a1: ANOVA and Dunnett test; a2: ANOVA and Dunnett test (log); a3: ANOVA and Dunnett test; a4: ANOVA and Dunnett test (rank); a5: ANOVA and Dunnett test (log); ^*∗*^*N* = 10/sex/group except at ^#^ where *N* = 9/group.

**Table 20:** Urinalysis—90 day dietary study^1^.

Parameter		Group 1 (0 ppm)		Group 2 (30,000 ppm)		Group 3 (60,000 ppm)		Group 4 (90,000 ppm)	
		M^*∗*^	F	M	F^#^	M	F	M^#^	F
Urine volume (ml)^a^	Mean ± SD	4 ± 3.36	1.62 ± 1.09	5.05 ± 2.98	1.52 ± 1.29	4.4 ± 1.32	3.93 ± 5.32	4.15 ± 3.54	3.26 ± 3.25
pH^a1^	Mean ± SD	6.4 ± 0.39	6 ± 0	6.45 ± 0.28	6.33 ± 0.5	6.2 ± 0.26	6.35 ± 0.47	6.39 ± 0.42	6.3 ± 0.35
Urine glucose (mg/dL)^a1^	Mean ± SD	0 ± 0	0 ± 0	0 ± 0*n*	0 ± 0*n*	0 ± 0*n*	0 ± 0*n*	0 ± 0*n*	0 ± 0*n*
Urine ketone (mmol/L)^a1^	Mean ± SD	5 ± 4.1	0 ± 0	8 ± 4.8	3.3 ± 5	16 ± 13.5^*∗∗*^	2 ± 2.6	17.8 ± 8.3^*∗∗∗*^	1.5 ± 2.4
Urine protein (mg/dL)^a1^	Mean ± SD	65 ± 36.9	56.5 ± 37.7	41 ± 31.7	51.7 ± 36.6	39.5 ± 33.4	57.5 ± 45.9	51.7 ± 36.6	46.5 ± 37.5
Specific gravity^a1^	Mean ± SD	1.026 ± 0.0052	1.03 ± 0	1.026 ± 0.0052	1.0272 ± 0.0057	1.026 ± 0.007	1.027 ± 0.0067	1.0267 ± 0.0071	1.029 ± 0.0032
Urobilinogen (EU/dL)^a1^	Mean ± SD	0.2 ± 0	0.2*n* ± 0	0.2 ± 0	0.2 ± 0*n*	0.2 ± 0	0.2 ± 0*n*	0.2 ± 0	0.2 ± 0*n*

^1^Urine obtained on Day 92 relative to start date. ^*∗*^*N* = 10/sex/group except at ^#^ where *N* = 9/group. M: male; F: female; LegH: leghemoglobin protein; SD: standard deviation. Statistical terms males: a: ANOVA and Dunnett test (log); a1: ANOVA and Dunnett test (rank); ^*∗∗*^ = *p* < 0.01; ^*∗∗∗*^ = *p* < 0.001; n-inappropriate for statistics; statistical terms females: a: ANOVA and Dunnett test (log); a1: ANOVA and Dunnett test (rank); *n*: inappropriate for statistics.

**Table 21:** Urinalysis—recovery phase.

Day(s) relative to start date (120)	Statistical term			Group 1 (0 ppm)		Group 4 (90,000 ppm)	
Parameter	M	F		M^*∗*^	F	M	F

Urine volume (ml)	a	a	Mean ± SD	4.2 ± 1.6	3.9 ± 2.9	3.7 ± 3.55	2.64 ± 2.58
pH	a1	a	Mean ± SD	6.4 ± 0.22	6.7 ± 0.76	6.3 ± 0.45	6.3 ± 0.76
Urine glucose (mg/dL)	a2	a1	Mean ± SD	0	0	0	0
Urine ketone (mmol/L)	a1	a1	Mean ± SD	5 ± 6.1	0 ± 0	2 ± 2.7	0 ± 0
Urine protein (mg/dL)	a	a2	Mean ± SD	98 ± 116.9	35 ± 38.4	95 ± 119.3	89 ± 124
Specific gravity	a2	a	Mean ± SD	1.028 ± 0.0027	1.021 ± 0.0089	1.028 ± 0.0045	1.027 ± 0.0045
Urobilinogen (EU/dL)	a2	a1	Mean ± SD	0.2 ± 0	0.2 ± 0	0.2 ± 0	0.2 ± 0

^*∗*^ *N* = 5/sex/group. M: male; F: female; LegH: leghemoglobin protein; SD: standard deviation. Statistical terms males: a: ANOVA and Dunnett test (log); a1: ANOVA and Dunnett test; a2: ANOVA and Dunnett test (rank). Statistical terms females: a: ANOVA and Dunnett; a1: ANOVA and Dunnett (rank), a2: ANOVA and Dunnett test (log).

**Table 22:** Thyroid hormone profile—90 day dietary study.

Day(s) relative to start date M (92), F (93)				Group 1 (0 ppm)		Group 2 (30,000 ppm)		Group 3 (60,000 ppm)		Group 4 (90,000 ppm)	
Parameter	Statistical term			M^#^	F	M	F	M	F	M	F
	M	F

TSH (ng/mL)	a	c	Mean ± SD	2.9548 ± 0.2143^#^	3.1087 ± 0.3449	2.75 ± 0.229	2.8384 ± 0.0967^*∗*^	3.2692 ± 0.1678^*∗∗*^	3.2968 ± 0.1975	3.2333 ± 0.2427^*∗*^	3.2296 ± 0.3588^##^
T4 (ng/mL)	a	d	Mean ± SD	49.2826 ± 3.0133	40.6217 ± 4.6983	50.5529 ± 5.6793	42.5739 ± 4.7898	45.6746 ± 5.5237	39.9237 ± 2.8535	44.4091 ± 3.1883	34.9737 ± 2.7091^*∗∗*^
T3 (ng/mL)	b	d	Mean ± SD	1.2892 ± 0.0356	1.611 ± 0.1804	1.4169 ± 0.0828	1.5371 ± 0.1623#	1.4500 ± 0.1323^*∗∗*^	1.502 ± 0.1838	1.4199 ± 0.0854^*∗∗*^	1.4716 ± 0.1557

^#^ *N* = 10/sex/group except for ^##^*N* = 9/sex/group. M: male; F: female; LegH: leghemoglobin protein; SD: standard deviation; TSH: thyroid stimulating hormone; T4: thyroxine; T3: triiodothyronine. Statistical terms males: a: ANOVA and Dunnett test; ^*∗*^ = *p* < 0.05; ^*∗∗*^ = *p* < 0.01; b: ANOVA and Dunnett test (rank); ^*∗∗*^ = *p* < 0.01. Statistical terms females: c: ANOVA and Dunnett test (log)^*∗*^ = *p* < 0.05; d: ANOVA and Dunnett (log); ^*∗∗*^ = *p* < 0.01.

**Table 23:** Thyroid hormone profile-recovery phase^1^.

Day(s) relative to start date (120)				Group 1 (0 ppm)		Group 4 (90,000 ppm)	
Parameter	Statistical term			M	F	M	F
	M	F

TSH (ng/mL)	a	b	Mean ± SD	3.7228 ± 0.229	3.6678 ± 0.2411	4.3748 ± 0.5011^*∗*^	3.796 ± 0.2964
T4 (ng/mL)	a	c	Mean ± SD	39.9522 ± 2.2033	34.9662 ± 3.2876	44.9994 ± 3.5658^*∗*^	48.2854 ± 18.2472
T3 (ng/mL)	a	b	Mean ± SD	1.3516 ± 0.1549	1.6262 ± 0.2093	1.4092 ± 0.1117	1.7252 ± 0.1338

^1^N = 5/sex/group. M: male; F: female; LegH: leghemoglobin protein; SD: standard deviation; TSH: thyroid stimulating hormone; T4: thyroxine; T3: triiodothyronine. Statistical terms male: a: ANOVA and Dunnett test; ^*∗*^ = *p* < 0.05; ^*∗∗*^ = *p* < 0.01. Statistical terms female: b: ANOVA and Dunnett test; c: ANOVA and Dunnett test (rank).

## Data Availability

Access to the underlying data included within this manuscript is restricted due to commercial confidentiality. Additional data are provided in a supplemental file.

## Supplementary Materials

Supplementary Materials Supplemental file: additional tabular data for the bacterial reverse mutation study, micronucleus study, and 28 day rat toxicology study are attached as a supplemental file.
