# Correction to “The Safety of Soy Leghemoglobin Protein Preparation Derived from *Pichia pastoris* Expressing a Soy Leghemoglobin Gene from *Glycine max*: *In Vitro* and *In Vivo* Studies”

**DOI:** 10.1155/jt/9862684

**Published:** 2026-01-15

T.F. Reyes, P. Agrawal, T. Chan, R. Green, and R.A. Matulka, “The Safety of Soy Leghemoglobin Protein Preparation Derived from *Pichia pastoris* Expressing a Soy Leghemoglobin Gene from *Glycine max*: *In Vitro* and *In Vivo* Studies,” *Journal of Toxicology*, 2023, https://doi.org/10.1155/2023/7398724.

In the article, there is an error in the batch numbers stated for the LegH Prep used in the *in vivo* and *in vitro* studies.

“The LegH Prep used in the in vivo and in vitro studies (batches LH20‐150‐160‐190FG‐301 and PP‐PGM2‐20‐061‐302) met internal specifications.”

should read as follows:

“The LegH Prep used in the *in vivo* and *in vitro* studies (batches LH20‐159‐160‐190FG‐301 and PP‐PGM2‐20‐061‐302) met internal specifications.”

Additionally, in the Materials and Methods section, the sentence beginning with

“Both strains were engineered to overexpress the LegH gene…”

should read as follows:

“The strains were engineered to overexpress the LegH gene…”

We apologize for these errors.

