# Supplementary material for: The Safety of Soy Leghemoglobin Protein Preparation Derived from Pichia pastoris Expressing a Soy Leghemoglobin Gene from Glycine max: In Vitro and In Vivo Studies
Source: J Toxicol. 2023 Oct 10;2023:7398724. doi: 10.1155/2023/7398724 (PMC10581837; doi:10.1155/2023/7398724)
Supplement: Supplementary Materials — Supplemental file: additional tabular data for the bacterial reverse mutation study, micronucleus study, and 28 day rat toxicology study are attached as a supplemental file. [file 7398724.f1.docx]

**Supplementary Information**

| **Table S1.** Results of the Pre-Experiment for the Bacterial Reverse Mutation Assay (Ames Test). | | | | | |
| --- | --- | --- | --- | --- | --- |
| **Substance** | **Dose (µg/plate)** | **TA98**  **Mutation Factor (Toxicity/Precipitation) *** | | **TA100**  **Mutation Factor (Toxicity/Precipitation) *** | |
|  |  |  |  |  |  |
|  |  | **Without S9** | **With S9** | **Without S9** | **With S9** |
| **Solvent Control**  **(A. dest)** | - | 1.0 | 1.0 | 1.0 | 1.0 |
| **4-NOPD** | 10.0 | 14.6 | - | - | - |
| **NaN3** | 10.0 | - | - | 4.1 | - |
| **2-AA** | 2.50 | - | 51.0 | - | 13.1 |
| **LegH Prep** | 3.16 | 1.8 | 1.0 | 0.9 | 1.1 |
|  | 10.0 | 1.1 | 1.0 | 0.9 | 1.0 |
|  | 31.6 | 0.9 | 0.9 | 1.0 | 1.0 |
|  | 100 | 0.9 | 0.9 | 0.9 | 1.0 |
|  | 316 | 0.9 | 0.9 | 1.0 | 0.9 |
|  | 1000 | 1.1 [P] | 1.0 [P] | 0.9 [P] | 1.0 [P] |
|  | 2500 | 0.8 [P] | 1.1 [P] | 1.0 [P] | 0.9 [P] |
|  | 5000 | 0.7 [P] | 1.1 [P] | 0.8 [P] | 0.9 [P] |
| *[toxicity / precipitation parameter]: B= Background lawn reduced; N= No background lawn; P= precipitation | | | | | |

| **Table S2.** Criteria of Validity Bacterial Reverse Mutation Assay. A test is considered acceptable if each bacterial strain demonstrates its typical responses to ampicillin (TA98, TA100, *E. col*i WP2 *uvrA* (pKM101)); and the negative control plates (A. dest.) with and without S9 mix are within the historical control data range (2017 – 2019 for TA tester strains; except December 2019 to May 2020 for *E. coli* WP2 uvrA). | | | | |
| --- | --- | --- | --- | --- |
| **STRAIN** | **-S9** | | **+S9** | |
|  | **min** | **max** | **min** | **max** |
| **TA98** | 14 | 61 | 15 | 60 |
| **TA100** | 44 | 143 | 60 | 154 |
| **TA1535** | 5 | 35 | 4 | 37 |
| **TA1537** | 3 | 35 | 5 | 41 |
| ***E. col*i WP2 *uvrA* (pKM101)** | 110 | 315 | 142 | 381 |
| S9 = rat liver metabolic activation system | | | | |

| **Table S3.** Dose levels– 14-day Dietary Palatability Study in Rats. | | | | |
| --- | --- | --- | --- | --- |
| **Group** | **No. Animals/ Group**  **M/F** | **Dietary Concentration**  **(ppm)^a^** | **Target Exposure (mg/kg/day) ^b^** | **% In Diet** |
| 1 | 5/5 | Group 1  (Basal Diet Control)  *0* | 0 | 0 |
| 2 | 5/5 | Group 2  *50,000 ppm* | 4167 | 5 |
| 3 | 5/5 | Group 3  *100,000 ppm* | 8333 | 10 |
| 4 | 5/5 | Group 4  *150,000 ppm* | 12,500 | 15 |
| ^a^ Concentration of Soy Leghemoglobin Preparation MF as mixed in the diet.  ^b^ Target mg/kg/day were estimated based on a 300-gram rat consuming 25 grams of diet per day | | | | |

Figure S1. Flow chart of the termination dates of the main and recovery phases of the 90-day toxicity study.

| **Table S4.** Dose levels– 90-day Dietary Feeding study in Rats with a 28-day Recovery Study. Freeze-dried LegH Prep concentration in the feed was adjusted every week based on average animal weight and food consumption to achieve the target exposure (mg/kg/day) of soy leghemoglobin active ingredient. | | | | |
| --- | --- | --- | --- | --- |
| **Main Study Groups** | | | | |
| **Group** | **No. Animals/ Group**  **(M/F)** | **Dietary Concentration (ppm)^a^** | **Target Exposure (mg/kg/day)** | **% In Diet** |
| 1 | 10/10 | Control (Basal Diet)  *0* | 0 | 0 |
| 2 | 10/10 | Group 1  *30,000* | 1875 | 3 |
| 3 | 10/10 | Group 2  *60,000* | 3750 | 6 |
| 4 | 10/10 | Group 3  *90,000* | 5625 | 9 |
| Recovery Groups | | | | |
| 1 | 5/5 | Control (Basal Diet)  0 | 0 | 0 |
| 4 | 5/5 | Group 3  90,000 | 5625 | 9 |
| ^a^ Concentration of Soy Leghemoglobin Preparation as mixed in the diet. | | | | |

| **Table S5: Diet Formulations (g/kg diet) for the 90-day subchronic rat study** | | | | |
| --- | --- | --- | --- | --- |
| **Group** | **Group 1 (Basal Diet (Control)** | **Group 2**  **(3% Soy Legh Prep)** | **6% Soy Leghemoglobin Preparation MF** | **9% Soy Leghemoglobin Preparation MF** |
| Soybean Oil (g) | 70 | 70.1 | 70.2 | 70.3 |
| Soy Leghemoglobin Prep (g) | 0 | 33 | 65 | 98 |
| L-Cysteine (g) | 3 | 3 | 3 | 3 |
| Casein (g) | 200 | 173.9 | 148.7 | 122.8 |
| Corn Starch (g) | 390.5 | 386.3 | 382.2 | 378 |
| Maltodextrin 10 (g) | 110 | 110 | 110 | 110 |
| Dextrose (g) | 150 | 150 | 150 | 150 |
| Cellulose BW200 (g) | 100 | 98.6 | 97.2 | 95.8 |
| Mineral Mix S10026(g) | 10 | 10 | 10 | 10 |
| DiCalcium Phosphate(g) | 13 | 13 | 13 | 13 |
| Calcium Carbonate (g) | 5.5 | 5.5 | 5.5 | 5.5 |
| Potassium Citrate, 1H_2_O (g) | 16.5 | 16.5 | 16.5 | 16.5 |
| Vitamin Mix V10001 (g) | 10 | 10 | 10 | 10 |
| Choline Bitartrate (g) | 2 | 2 | 2 | 2 |
| **Total** (g) | **1080.5** | **1082.5** | **1084.4** | **1086.6** |

| **Table S6. Test for Cytotoxicity, without and with metabolic activation** in the Pre-Experiment– *in vitro* Micronucleus study with human lymphocytes. The cytokinesis block proliferation index (CBPI) was used to calculate the cytostasis (cytotoxicity). The concentrations used in the main experiment were based on the results obtained in the pre-experiment. | | | | | |
| --- | --- | --- | --- | --- | --- |
| **Dose Group** | **Concentration (µg/mL)** | **CBPI** | **Relative Cell Growth (%)** | **Cytostasis (%)** | **Precipitate**  **(+/-)** |
| **Without metabolic activation** | | | | | |
| **C** | 0 | 1.49 | 100 | 0 | - |
| **1** | 10 | 1.45 | 91 | 9 | - |
| **2** | 20 | 1.38 | 79 | 21 | - |
| **3** | 39 | 1.40 | 82 | 18 | - |
| **4** | 78 | 1.45 | 92 | 8 | - |
| **5** | 156 | 1.54 | 111 | 0 | - |
| **6** | 312.5 | 1.54 | 111 | 0 | + |
| **7** | 625 | 1.45 | 92 | 8 | + |
| **8** | 1250 | Ne | Ne | Ne | + |
| **9** | 5000 | 1.37 | 76 | 24 | + |
| **10** |  | ne | ne | Ne | + |
| **With metabolic activation** | | | | | |
| **C** | 0 | 1.52 | 100 | 0 | - |
| **1** | 10 | 1.50 | 95 | 5 | - |
| **2** | 20 | 1.57 | 109 | 0 | - |
| **3** | 39 | 1.47 | 90 | 10 | - |
| **4** | 78 | 1.40 | 77 | 23 | - |
| **5** | 156 | 1.40 | 76 | 24 | - |
| **6** | 312.5 | 1.62 | 119 | 0 | + |
| The CBPI was determined in 500 cells per culture of each test group.  The relative values of the CBPI are related to the negative controls.  C: Negative control (culture medium)  CBPI: Cytokinesis Block Proliferation Index = ((c1x1) + (c2 x 2) + (cx x 3))/n  Relative cell growth: 100 x (CBPI test conc – 1)/CBPI control -1))  C1: mononucleate cells  C2: binucleate cells  Cx: multinucleate cells  N: total number of cells  Ne: Not evaluable  Cytostasis (%) = 100 – Relative Cell Growth (%)  The cytostasis is defined 0, when the relative cell growth exceeds 100% | | | | | |

| **Table** **S7.** Summary of mean daily body weight gain (g/day)- 14-Day Study^a,b^ | | | | | | | | | |
| --- | --- | --- | --- | --- | --- | --- | --- | --- | --- |
| **Day(s) Relative to Start Date** | | **Group 1**  **(0 ppm)** | | **Group 2**  **(50000 ppm)** | | **Group 3**  **(100,000 ppm)** | | **Group 4**  **(150000 ppm)** | |
|  |  | **M#** | **F** | **M** | **F** | **M** | **F** | **M** | **F** |
| **0→3** | Mean±  SD | 9.40±0.92 | 3.73±0.83 | 10.53±0.84 | 4.40±2.15 | 8.80±0.77 | 3.20±1.28 | 7.67**±0.62 | 3.13±0.84 |
| **3→7** | Mean±  SD | 9.25±0.90 | 2.45±1.10 | 8.65±0.95 | 2.20±1.19 | 7.25*±1.58 | 2.75±0.47 | 6.65**±0.88 | 1.70±1.04 |
| **7→10** | Mean±  SD | 7.60±2.13 | 1.87±1.56 | 7.13±1.04 | 3.40±2.07 | 8.20±1.24 | 3.33±2.36 | 7.33±1.13 | 2.93±1.19 |
| **10→14** | Mean±  SD | 5.85±0.96 | 1.45±1.37 | 6.05±0.86 | 1.55±1.59 | 5.25±0.50 | 1.80±0.54 | 4.50*±0.50 | 0.30±1.20 |
| ^a^ Statistical analysis Males: One Way Repeat Anova & Dunnett* = p<0.05; ** p<0.01  ^b^ Statistical analysis Females: One Way Repeat Anova & Dunnett  F = female; M = male; SD = standard deviation. #N=5/sex/group | | | | | | | | | |

| [**Table**](https://docs.google.com/document/d/1LZHBoS3DsLYeJZpFJ-K6ZIIC4N_vRXrVVEYs6H_U2Zk/edit) **S8.** Summary of daily food consumption (g/day)- 14-Day Study* | | | | | | | | | |
| --- | --- | --- | --- | --- | --- | --- | --- | --- | --- |
| **Day(s) Relative to Start Date** | | **Group 1**  **(0 ppm)** | | **Group 2**  **(50000 ppm)** | | **Group 3**  **(100,000 ppm)** | | **Group 4**  **(150000 ppm)** | |
|  |  | **M#** | **F** | **M** | **F** | **M** | **F** | **M** | **F** |
| **0→3** | Mean±SD | 23.47±2.14 | 17±1.39 | 23.60±1.01 | 16.87±2.12 | 22.07±2.05 | 16.27±2.01 | 20.27±1.09 | 16.07±1.23 |
| **3→7** | Mean±SD | 26.15±2.68 | 18.55±2.01 | 26.05±0.93 | 17.10±2.53 | 24.05±2.22 | 19.05±4.43 | 23.50±0.68 | 16.85±1.43 |
| **7→10** | Mean±SD | 25.47±2.67 | 15.80±0.96 | 25.93±1.71 | 17.27±1.83 | 24.67±2.39 | 17.53±2.04 | 23.33±1.13 | 17.80±3.32 |
| **10→14** | Mean±SD | 26.80±2.67 | 17.35±2.21 | 26.45±1.60 | 16.85±1.91 | 24.90±2.28 | 18±1.72 | 24±1.74 | 16±1.46 |
| *One Way Repeat Anova & Dunnett  F = female; M = male; SD = standard deviation. # N=5/sex/group | | | | | | | | | |

| [**Table**](https://docs.google.com/document/d/1LZHBoS3DsLYeJZpFJ-K6ZIIC4N_vRXrVVEYs6H_U2Zk/edit) **S9.** Summary of mean food efficiency^a^-14-Day Study* | | | | | | | | | |
| --- | --- | --- | --- | --- | --- | --- | --- | --- | --- |
| **Day(s) Relative to Start Date** | | **Group 1**  **(0 ppm)** | | **Group 2**  **(50000 ppm)** | | **Group 3**  **(100,000 ppm)** | | **Group 4**  **(150000 ppm)** | |
|  |  | **M#** | **F** | **M** | **F** | **M** | **F** | **M** | **F** |
| **0→3** | Mean±  SD | 0.401±0.032 | 0.250±0.106 | 0.447±0.045 | 0.250±0.106 | 0.400±0.027 | 0.194±0.063 | 0.378±0.025 | 0.195±0.048 |
| **3→7** | Mean±  SD | 0.356±0.044 | 0.123±0.055 | 0.333±0.042 | 0.123±0.055 | 0.300±0.045 | 0.150±0.042 | 0.284±0.042 | 0.099±0.056 |
| **7→10** | Mean±  SD | 0.297±0.075 | 0.199±0.115 | 0.274±0.028 | 0.199±0.115 | 0.332±0.025 | 0.181±0.103 | 0.314±0.048 | 0.164±0.052 |
| **10→14** | Mean±  SD | 0.218±0.028 | 0.091±0.097 | 0.229±0.030 | 0.091±0.097 | 0.212±0.027 | 0.102±0.036 | 0.188±0.024 | 0.014±0.076 |
| *One Way Repeat Anova & Dunnett  ^a^ Food efficiency= Mean Daily Body Weight Gain/Mean Daily Food Consumption  F = female; M = male; SD = standard deviation. #N=5/sex/group | | | | | | | | | |

| **Table S10.** Summary of Mean Daily Food Consumption (g/rat/day) --90-Day Dietary Study ^a, *^ | | | | | | | | | |
| --- | --- | --- | --- | --- | --- | --- | --- | --- | --- |
| **Day(s) Relative to Start Date** |  | **Group 1**  **(0 ppm)** | | **Group 2**  **(30,000 ppm)** | | **Group 3**  **(60,000 ppm)** | | **Group 4**  **(90,000 ppm)** | |
| **0 → 7** |  | M | F | M | F | M | F | M | F |
|  | Mean±SD | 24.40±1.18 | 17.63±1.79 | 24.76±1.85 | 17.79±1.89 | 25.16±2.43 | 17.86±2.79 | 24.54±1.68 | 17.16±1.76 |
|  | N | 15 | 15 | 10 | 10 | 10 | 10 | 15 | 15 |
| **10 → 14** | Mean±SD | 25.6±1.68 | 20.7±6 | 25.08±2.32 | 19.53±3.94 | 25.78±2.44 | 18.65±2.58 | 25.18±2.06 | 18.88±2.64 |
|  | N | 15 | 15 | 10 | 10 | 10 | 10 | 15 | 15 |
| **14 → 21** | Mean±SD | 25.44±1.96 | 20.7±3.71 | 25.67±2.41 | 19.64±2.58 | 26.04±2.54 | 18.97±2.69 | 25.48±2.27 | 19.2±3 |
|  | N | 15 | 15 | 10 | 10 | 10 | 10 | 15 | 15 |
| **21 → 27** | Mean±SD | 25.23±1.91 | 20.12±3.36 | 25.8±3.08 | 19.43±2.71 | 26.22±2.96 | 19.75±3.42 | 25.76±2.23 | 19.85±4.04 |
|  | N | 15 | 15 | 10 | 10 | 10 | 10 | 15 | 15 |
| **27 → 34** | Mean±SD | 26.1±2.16 | 21.41±3.13 | 26.46±3.48 | 19.97±3.57 | 27.1±2.82 | 19.86±3.71 | 27.17±2.4 | 19.97±3.4 |
|  | N | 15 | 15 | 10 | 10 | 10 | 10 | 15 | 15 |
| **34 → 41** | Mean±SD | 26.09±2.12 | 21.54±4.04 | 27.11±3.33 | 20.16±4.13 | 27.69±2.7 | 20.96±4.59 | 27.13±2.48 | 20.01±2.38 |
|  | N | 15 | 15 | 10 | 10 | 10 | 10 | 15 | 15 |
| **41 → 49** | Mean±SD | 25.89±1.7 | 19.6±3.87 | 26.59±3.12 | 19.18±3.71 | 26.9±2.54 | 19.6±4.48 | 26.29±2.4 | 19.34±3.02 |
|  | N | 15 | 15 | 10 | 10 | 10 | 10 | 15 | 15 |
| **49 → 56** | Mean±SD | 25.64±1.51 | 19.51±3.93 | 26.67±3.03 | 18.93±3.38 | 26.16±2.32 | 19±4.47 | 25.7±2.52 | 18.4±2.77 |
|  | N | 15 | 15 | 10 | 10 | 10 | 10 | 15 | 15 |
| **56 → 63** | Mean±SD | 24.54±1.6 | 18.71±4.87 | 25.06±2.29 | 17.96±3.62 | 25.6±3.43 | 18.67±3.51 | 24.56±2.57 | 18.01±2.43 |
|  | N | 15 | 15 | 10 | 10 | 10 | 10 | 15 | 15 |
| **63 → 70** | Mean±SD | 24.24±1.37 | 20.21±2.9 | 26.39±2.53 | 18±3.1 | 25.09±2.48 | 17.74±2.35 | 24.85±2.33 | 18.6±2.25 |
|  | N | 15 | 15 | 10 | 10 | 10 | 10 | 15 | 15 |
| **70 → 77** | Mean±SD | 24.51±2.01 | 20.01±3.32 | 26.29±3.05 | 19.33±3.81 | 25.66±2.1 | 19±3.94 | 24.92±2.03 | 18.6±2.64 |
|  | N | 15 | 15 | 10 | 10 | 10 | 10 | 15 | 15 |
| **77 → 84** | Mean±SD | 23.8±1.99 | 20.56±4.47 | 25.46±2 | 18.39±4.17 | 23.27±1.99 | 17.56±2.62 | 23.54±2.32 | 18.13±2.83 |
|  | N | 15 | 15 | 10 | 10 | 10 | 10 | 15 | 15 |
| **84 → 91** | Mean±SD | 23.51±1.67 | 18.21±4.73 | 24.59±1.98 | 17.63±2.46 | 23.94±2.14 | 18.29±3.27 | 24.13±2.69 | 18.43±2.54 |
|  | N | 15 | 15 | 10 | 10 | 10 | 10 | 15 | 15 |
| ^a^One Way Repeat Anova & Dunnett  ^*^N= 10/sex/group  M, Male, F, Female, LegH, leghemoglobin protein, SD, standard deviation | | | | | | | | | |

| **Table S11.** Summary of Mean Daily Food Consumption (g/rat/day) –90-day Study-Recovery Phase ^a, b^ | | | | | |
| --- | --- | --- | --- | --- | --- |
| **Day(s) Relative to Start Date** |  | **Group 1**  **(0 ppm)** | | **Group 4**  **(90,000 ppm)** | |
| **0 → 7** |  | M | F | M | F |
|  | Mean±SD | 24.03±2.1 | 18.54±1.26 | 24.33±2.08 | 16.57±2.13 |
|  |  |  |  |  |  |
| **10 → 14** | Mean±SD | 25.15±2.38 | 20.2±1.99 | 24±2.04 | 17.15±1.08 |
|  |  |  |  |  |  |
| **14 → 21** | Mean±SD | 25.97±1.98 | 20.51±1.94 | 25.71±2.68 | 17.43±1.63 |
|  |  |  |  |  |  |
| **21 → 27** | Mean±SD | 26.3±2.72 | 21.93±2.64 | 26.13±2.85 | 17.6±2.13 |
|  |  |  |  |  |  |
| **27 → 34** | Mean±SD | 27.14±3.19 | 22.43±2.82 | 27.34±2.89 | 19±3.21 |
|  |  |  |  |  |  |
| **34 → 41** | Mean±SD | 27.43±3.03 | 21.29±3.32 | 27.46±3.01 | 18.26±2.78 |
|  |  |  |  |  |  |
| **41 → 49** | Mean±SD | 27.4±2.58 | 20.35±3.3 | 26.43±3.3 | 16.93±1.75 |
|  |  |  |  |  |  |
| **49 → 56** | Mean±SD | 26.89±2.5 | 17.51±1.7 | 26.34±3.3 | 17.83±2.15 |
|  |  |  |  |  |  |
| **56 → 63** | Mean±SD | 26.06±2.38 | 18.8±3.57 | 25.06±3.51 | 16.69±1.34 |
|  |  |  |  |  |  |
| **63 → 70** | Mean±SD | 25.69±2.01 | 16.89±0.49 | 24.6±2.84 | 16.09±1.08 |
|  |  |  |  |  |  |
| **70 → 77** | Mean±SD | 26.29±1.71 | 19.86±5.13 | 25.86±2.18 | 16.09±1.16 |
|  |  |  |  |  |  |
| **77 → 84** | Mean±SD | 24.23±1.06 | 18.66±4.1 | 24.69±3.06 | 16.29±1.75 |
|  |  |  |  |  |  |
| **84 → 91** | Mean±SD | 23.86±1.79 | 17.77±2.65 | 25.03±3.1 | 16.63±2.42 |
|  |  |  |  |  |  |
| **91 → 98** | Mean±SD | 25.63±2.04 | 17.71±1.04 | 26.4±2.15 | 18.46±1.27 |
|  |  |  |  |  |  |
| **98 → 105** | Mean±SD | 24.17±1.72 | 18.31±1.83 | 24.11±1.93 | 16.49±1.47 |
|  |  |  |  |  |  |
| **105 → 112** | Mean±SD | 25.71±2.09 | 17.89±1.28 | 25.29±2.69 | 17.26±1.48 |
|  |  |  |  |  |  |
| **112 → 119** | Mean±SD | 25.11±2.01 | 17.66±2.13 | 25.09±2.03 | 17.4±2.98 |
| ^a^One Way Repeat Anova & Dunnett  ^b^N= 5/sex/group  Abbreviations: M, Male, F, Female, LegH, leghemoglobin protein, SD, standard deviation | | | | | |

| **Table S12.** Summary of Mean Food Efficiency--90-Day Dietary Study ^a,b.c^ | | | | | | | | | |
| --- | --- | --- | --- | --- | --- | --- | --- | --- | --- |
| **Day(s) Relative to Start Date** |  | **Group 1**  **(0 ppm)** | | **Group 2**  **(30,000 ppm)** | | **Group 3**  **(60,000 ppm)** | | **Group 4**  **(90,000 ppm)** | |
| **0 → 7** |  | M | F | M | F | M | F | M | F |
|  | Mean±SD | 0.351±0.035 | 0.247±0.044 | 0.371±0.052 | 0.282±0.033 | 0.38±0.033 | 0.236±0.034 | 0.349±0.053 | 0.238±0.033 |
| **14 → 21** | Mean±SD | 0.22±0.023 | 0.152±0.076 | 0.231±0.028 | 0.132±0.041 | 0.233±0.032 | 0.142±0.045 | 0.22±0.03 | 0.177±0.054 |
| **21 → 27** | Mean±SD | 0.206±0.018 | 0.147±0.043 | 0.198±0.051 | 0.15±0.066 | 0.214±0.026 | 0.124±0.07 | 0.22±0.028 | 0.12±0.053 |
| **27 → 34** | Mean±SD | 0.15±0.019 | 0.045±0.048 | 0.14±0.024 | 0.095±0.038 | 0.16±0.015 | 0.096±0.05 | 0.165±0.02 | 0.103±0.049 |
| **34 → 41** | Mean±SD | 0.167±0.013 | 0.111±0.03 | 0.146±0.029 | 0.076±0.031 | 0.154±0.03 | 0.087±0.043 | 0.143±0.017 | 0.116±0.033 |
| **41 → 49** | Mean±SD | 0.158±0.037 | 0.063±0.049 | 0.151±0.014 | 0.08±0.021 | 0.153±0.022 | 0.077±0.018 | 0.148±0.024 | 0.084±0.05 |
| **49 → 56** | Mean±SD | 0.11±0.013 | 0.007±0.084 | 0.113±0.016 | 0.059±0.034 | 0.107±0.025 | 0.048±0.052 | 0.102±0.039 | 0.032±0.056 |
| **56 → 63** | Mean±SD | 0.093±0.029 | 0.067±0.059 | 0.074±0.021 | 0.03±0.041 | 0.073±0.025 | 0.046±0.025 | 0.09±0.032 | 0.066±0.05 |
| **63 → 70** | Mean±SD | 0.096±0.019 | 0.05±0.062 | 0.087±0.025 | 0.056±0.026 | 0.09±0.026 | 0.054±0.035 | 0.096±0.027 | 0.063±0.037 |
| **70 → 77** | Mean±SD | 0.079±0.031 | 0.091±0.039 | 0.087±0.028 | 0.071±0.034 | 0.072±0.034 | 0.061±0.047 | 0.089±0.027 | 0.068±0.043 |
| **77 → 84** | Mean±SD | 0.089±0.023 | 0.052±0.061 | 0.08±0.02 | 0.016±0.034 | 0.068±0.029 | 0.031±0.072 | 0.087±0.02 | 0.02±0.066 |
| **84 → 91** | Mean±SD | 0.062±0.031 | 0.018±0.05 | 0.062±0.022 | 0.041±0.065 | 0.064±0.022 | 0.024±0.062 | 0.085±0.025 | 0.033±0.065 |
| **Marginal** | Mean±SD | 0.148±0.082 | 0.087±0.084 | 0.145±0.089 | 0.091±0.079 | 0.147±0.093 | 0.086±0.074 | 0.15±0.082 | 0.093±0.078 |
|  | N | 120 | 120 | 120 | 120 | 120 | 120 | 180 | 120 |
| ^a^One Way Repeat Anova & Dunnett  ^b^ Food efficiency= Calculated Daily Body Weight Gain        Calculated Daily Food Consumption  ^c^ N= 10/sex/group  Abbreviations: M, Male, F, Female, LegH, leghemoglobin protein, SD, standard deviation | | | | | | | | | |

| **Table S13.** Summary of Mean Food Efficiency (N=5/sex/group)—Recovery Group ^a,b^ | | | | | |
| --- | --- | --- | --- | --- | --- |
| **Day(s) Relative to Start Date** |  | **Group 1**  **(0 ppm)** | | **Group 4**  **(90,000 ppm)** | |
| **0 → 7** |  | M | F | M | F |
|  | Mean±SD | 0.373±0.027 | 0.287±0.023 | 0.339±0.067 | 0.216±0.07 |
| **14 → 21** | Mean±SD | 0.25±0.018 | 0.157±0.035 | 0.245±0.029 | 0.14±0.041 |
| **21 → 27** | Mean±SD | 0.238±0.033 | 0.117±0.035 | 0.229±0.017 | 0.119±0.049 |
| **27 → 34** | Mean±SD | 0.176±0.028 | 0.098±0.023 | 0.165±0.026 | 0.14±0.031 |
| **34 → 41** | Mean±SD | 0.177±0.013 | 0.059±0.022 | 0.145±0.016 | 0.067±0.081 |
| **41 → 49** | Mean±SD | 0.173±0.015 | 0.052±0.042 | 0.155±0.022 | 0.083±0.027 |
| **49 → 56** | Mean±SD | 0.127±0.012 | 0.06±0.045 | 0.12±0.011 | 0.088±0.06 |
| **56 → 63** | Mean±SD | 0.084±0.029 | 0.038±0.038 | 0.101±0.039 | 0.057±0.06 |
| **63 → 70** | Mean±SD | 0.109±0.014 | 0.064±0.034 | 0.097±0.028 | 0.044±0.043 |
| **70 → 77** | Mean±SD | 0.101±0.014 | 0.065±0.022 | 0.085±0.029 | 0.051±0.025 |
| **77 → 84** | Mean±SD | 0.053±0.045 | 0.027±0.024 | 0.096±0.013 | 0.019±0.027 |
| **84 → 91** | Mean±SD | 0.077±0.015 | 0.048±0.051 | 0.077±0.02 | 0.034±0.057 |
| **91 → 98** | Mean±SD | 0.068±0.014 | 0.065±0.041 | 0.07±0.02 | 0.083±0.049 |
| **98 → 105** | Mean±SD | 0.052±0.017 | 0.037±0.032 | 0.045±0.032 | 0.022±0.049 |
| **105 → 112** | Mean±SD | 0.072±0.042 | 0.057±0.051 | 0.065±0.018 | 0.065±0.039 |
| **112 → 119** | Mean±SD | 0.057±0.034 | 0.067±0.07 | 0.066±0.008 | 0.062±0.067 |
| **Marginal** | Mean±SD | 0.137±0.091 | 0.081±0.072 | 0.131±0.082 | 0.081±0.068 |
| ^a^1 Way Repeat Anova & Dunnett  ^b^ Food efficiency= Calculated Daily Body Weight Gain/Calculated Daily Food Consumption  Abbreviations: M, Male, F, Female, LegH, leghemoglobin protein, SD, standard deviation | | | | | |

| [**Table S14.**](https://docs.google.com/document/d/1LZHBoS3DsLYeJZpFJ-K6ZIIC4N_vRXrVVEYs6H_U2Zk/edit) Summary of Mean Relative Organ-to-body Weights (g) –90-Day Dietary Study | | | | | | | | | | | |
| --- | --- | --- | --- | --- | --- | --- | --- | --- | --- | --- | --- |
| **LegH dose levels** | **Statistical Term** | |  | **Group 1**  **(0 ppm)** | | **Group 2 (30,000 ppm)** | | **Group 3 (60,000 ppm)** | | **Group 4**  **(90,000 ppm)** | |
| **Parameter** | M | F |  | M# | F | M | F | M | F | M | F |
| **Adrenal/TBW (Ratio)** | a | a | Mean±SD | 0.1021±  0.0301 | 0.2027±  0.0349 | 0.0998±  0.023 | 0.1996±  0.0364 | 0.1043±  0.0261 | 0.2319±  0.038 | 0.092±  0.0237 | 0.2151±  0.0342 |
|  |  |  |  |  |  |  |  |  |  |  |  |
| **Brain/TBW (Ratio)** | a | a | Mean±SD | 3.941±  0.175 | 6.602±  1.063 | 3.78±  0.388 | 6.484±  0.632 | 3.847±  0.366 | 6.583±  0.603 | 3.879±  0.239 | 6.458±  0.819 |
|  |  |  |  |  |  |  |  |  |  |  |  |
| **Epididymis/ TBW (Ratio)** | a | - | Mean±SD | 2.6253±  0.2339 | - | 2.7169±  0.2619 | - | 2.7345±  0.3059 | - | 2.5785±  0.3439 | - |
|  |  |  |  |  |  |  |  |  |  |  |  |
| **Heart/TBW (Ratio)** | a | c | Mean±SD | 2.808±  0.166 | 3.285±  0.3 | 2.714±  0.128 | 3.141±  0.26 | 2.716±  0.217 | 3.276±  0.186 | 2.676±  0.29 | 3.333±  0.49 |
|  |  |  |  |  |  |  |  |  |  |  |  |
| **Kidneys/TBW (Ratio)** | f | b | Mean±SD | 5.812±  0.6 | 6.721±  0.608 | 6.34±  0.581 | 6.419±  0.407 | 6.293±  0.724 | 6.71±  0.405 | 6.036±  0.487 | 7.062±  1.02 |
|  |  |  |  |  |  |  |  |  |  |  |  |
| **Liver/TBW (Ratio)** | f | a | Mean±SD | 23.792±  1.933 | 26.358±  2.605 | 24.902±  2.209 | 24.299±  1.328 | 25.092±  1.902 | 25.182±  1.992 | 23.131±  2.692 | 26.488±  2.755 |
|  |  |  |  |  |  |  |  |  |  |  |  |
| **Pituitary/TBW (Ratio)** | a | c | Mean±SD | 0.0027±  0.0005 | 0.0093±  0.0037 | 0.0034±  0.0009 | 0.0075±  0.0023 | 0.0027±  0.0008 | 0.009±  0.0036 | 0.0031±  0.001 | 0.0083±  0.0031 |
|  |  |  |  |  |  |  |  |  |  |  |  |
| **Pro, SV, CG (combined)/ TBW (Ratio)** | a | - | Mean±SD | 0.006±  0.001 | - | 0.006±  0.001 | - | 0.005±  0.001 | - | 0.006±  0.001 | - |
|  |  |  |  |  |  |  |  |  |  |  |  |
|  |  |  | N | 10 | - | 9## | - | 10 | - | 10 | - |
| **Spleen/TBW (Ratio)** | a | a | Mean±SD | 1.566±  0.145 | 1.814±  0.308 | 1.559±  0.114 | 1.539±  0.137 | 1.427±  0.197 | 1.664±  0.306 | 1.43±  0.277 | 1.643±  0.286 |
|  |  |  |  |  |  |  |  |  |  |  |  |
| **Testes/TBW (Ratio)** | f | - | Mean±SD | 6.154±  0.741 | - | 6.245±  0.602 | - | 6.221±  0.624 | - | 6.293±  0.585 | - |
|  |  |  |  |  |  |  |  |  |  |  |  |
| **Thymus/TBW (Ratio)** | e | c | Mean±SD | 0.5904±  0.1547 | 1.0478±  0.3276 | 0.4483±  0.077* | 0.8559±  0.2851 | 0.5467±  0.1056 | 0.7308±  0.1056* | 0.4462±  0.118* | 0.7896±  0.1872 |
|  |  |  |  |  |  |  |  |  |  |  |  |
| **Thyroid-Parathyroid/ TBW (Ratio)** | e | b | Mean±SD | 0.4464±  0.12201 | 0.75609±  0.30197 | 0.55585±  0.18021 | 0.81656±  0.23839 | 0.52261±  0.09894 | 0.85358±  0.0576 | 0.59005±  0.08934 | 0.9797  0.17293± |
|  |  |  |  |  |  |  |  |  |  |  |  |
| **Ovaries with Oviducts/ TBW (Ratio)** | - | a | Mean±SD | - | 0.3571±  0.0559 | - | 0.3377±  0.0708 | - | 0.3177±  0.1 | - | 0.3687±  0.069 |
|  |  |  |  |  |  |  |  |  |  |  |  |
| **Uterus/TBW (Ratio)** | - | f | Mean±SD | - | 2.263±  0.736 | - | 2.103±  0.749 | - | 2.279±  0.532 | - | 2.012±  0.708 |
|  |  |  |  |  |  |  |  |  |  |  |  |
| N= 10/sex/group unless indicated as ## N = 9/group.  Abbreviations: M, Male, F, Female, LegH, leghemoglobin protein, SD, standard deviation  Statistical Terms: a, Anova & Dunnett; b, Kruskal-Wallis & Dunn, c, Anova & Dunnett (Log): * = p < 0.05, d, Anova & Dunnett ** = p < 0.01; e. Anova & Dunnett* = p < 0.05; f, Anova & Dunnett (Log); | | | | | | | | | | | |

| [**Table**](https://docs.google.com/document/d/1LZHBoS3DsLYeJZpFJ-K6ZIIC4N_vRXrVVEYs6H_U2Zk/edit) **S15.** Summary of Mean Relative Organ-to-body Weights (g) –Recovery Phase | | | | | | | |
| --- | --- | --- | --- | --- | --- | --- | --- |
| **LegH Dose Levels** | **Statistical Term** | | | **Group 1**  **(0 ppm)** | | **Group 4**  **(90,000 ppm)** | |
| **Parameter** | **M** | **F** |  | M* | F | M | F |
| **Adrenal/TBW (Ratio)** | e | a | Mean±SD | 0.0738±0.0284 | 0.1993±0.0365 | 0.0917±0.0145 | 0.2073±0.585 |
|  |  |  |  |  |  |  |  |
| **Brain/TBW (Ratio)** | e | a | Mean±SD | 3.518±0.345 | 6.006±0.652 | 3.516±0.324 | 6.103±0.445 |
|  |  |  |  |  |  |  |  |
| **Epididymis/TBW (Ratio)** | e | - | Mean±SD | 2.3404±0.2462 | - | 2.8179±0.3609* | - |
|  |  |  |  |  |  |  |  |
| **Heart/TBW (Ratio)** | e | c | Mean±SD | 2.615±0.311 | 3.175±0.21 | 2.582±0.141 | 3.025±0.252 |
|  |  |  |  |  |  |  |  |
| **Kidneys/TBW (Ratio)** | b | b | Mean±SD | 5.392±0.356 | 6.286±0.668 | 4.629±2.509 | 6.038±0.41 |
|  |  |  |  |  |  |  |  |
| **Liver/TBW (Ratio)** | e | a | Mean±SD | 22.674±2.414 | 25.462±2.292 | 24.199±1.347 | 23.529±1.35 |
|  |  |  |  |  |  |  |  |
| **Pituitary/TBW (Ratio)** | c | c | Mean±SD | 0.0034±0.0008 | 0.0073±0.0027 | 0.0026±0.0003* | 0.007±0.0015 |
|  |  |  |  |  |  |  |  |
| **Pro, SV, CG (combined)/TBW (Ratio)** | b | - | Mean±SD | 0.005±0 | - | 0.006±0.002 | - |
|  |  |  |  |  |  |  |  |
| **Spleen/TBW (Ratio)** | c | a | Mean±SD | 1.425±0.241 | 1.55±0.264 | 1.42±0.184 | 1.666±0.146 |
|  |  |  |  |  |  |  |  |
| **Testes/TBW (Ratio)** | e | - | Mean±SD | 5.721±0.404 | - | 6.337±0.904 | - |
|  |  |  |  |  |  |  |  |
| **Thymus/TBW (Ratio)** | a | c | Mean±SD | 0.3361±0.0696 | 0.6767±0.1184 | 0.3061±0.1146 | 0.5967±0.111 |
|  |  |  |  |  |  |  |  |
| **Thyroid-Parathyroid/TBW (Ratio)** | a | b | Mean±SD | 0.59223±0.13084 | 1.13666±0.16291 | 0.68185±0.08464 | 1.01281±0.1466 |
|  |  |  |  |  |  |  |  |
| **Ovaries with Oviducts/TBW (Ratio)** | - | a | Mean±SD | - | 0.2831±0.0309 | - | 0.4004±0.0696** |
|  |  |  |  |  |  |  |  |
| **Uterus/TBW (Ratio)** | - | f | Mean±SD | - | 2.179±0.197 | - | 1.925±0.514 |
|  |  |  |  |  |  |  |  |
| *N= 5/sex/group  Abbreviations: M, Male, F, Female, LegH, leghemoglobin protein, SD, standard deviation  Statistical Terms: a, Anova & Dunnett; b, Kruskal-Wallis & Dunn, c, Anova & Dunnett (Log): * = p < 0.05, d, Anova & Dunnett ** = p < 0.01; e. Anova & Dunnett* = p < 0.05; f, Anova & Dunnett (Log). | | | | | | | |

| **Table S16.** Summary of Estrous Cycle Stage Distribution (Animal #) –90-Day Dietary Study ^a^ and Recovery Phase ^b^ | | | | |
| --- | --- | --- | --- | --- |
|  | **90-Day study: Main Phase^a^** | | **90-Day study: Recovery Phase^b^** | |
| **LegH Dose Levels** | **Group 1**  **(0 ppm)** | **Group 4**  **(90,000 ppm)** | **Group 1**  **(0 ppm)** | **Group 4**  **(90,000 ppm)** |
| **Diestrus** | 3 | 5 | 1 | 2 |
| **Estrus** | 6 | 4 | 3 | 2 |
| **Proestrus** | 1 | 1 | 1 | 1 |
| **Metestrus** | 0 | 0 | 0 | 0 |
| ^a^N=10/sex/group, Day 93  ^b^N= 5/sex/group, Day 120  LegH =Leghemoglobin protein preparation; ppm=parts per million | | | | |
